# Supplementary material for: Preparation of a Montmorillonite-Modified Chitosan Film-Loaded Palladium Heterogeneous Catalyst and its Application in the Preparation of Biphenyl Compounds
Source: Molecules. 2022 Dec 16;27(24):8984. doi: 10.3390/molecules27248984 (PMC9782881; doi:10.3390/molecules27248984)
Supplement: Supplementary file 1 [file molecules-27-08984-s001.zip › molecules-2051673-supplementary.pdf]

Table of Contents

1. Table **S1** Mixing ratio of CS/PVA/MMT blended film	S1
2. Table **S2** Mechanical properties of CS, PVA, and CS/PVA blended film	S1
3. Figure **S1** Adsorption of Pd^{2+} by CS/PVA/MMT is consistent with the pseudo–frist–order kinetic model	S2
4. Figure **S2** Adsorption of Pd^{2+} by CS/PVA/MMT is consistent with the Weber Morris model model	S3
5. Figure **S3** FTIR spectra of CS/PVA/MMT @Pd	S4
6. Figure **S4** X–ray diffraction patterns of CS/PVA/MMT @Pd (after one reaction)	S5
7. Characterization data for products (**3a– 3ab**)	S6
8. Fig. **S5–S32** ^{1}H NMR and ^{13}C NMR spectra of products (**3a– 3ab**)	S12

Table S1. Mixing ratio of CS/PVA/MMT blended film.

Entry	1	2	3	4	5
2% CS/ mL	10	10	10	10	10
2% PVA/ mL	10	10	10	10	10
MMT/(g)	0.020	0.040	0.060	0.080	0.100
H_2O/ mL	30	30	30	30	30

Table S2. Mechanical properties of CS, PVA, and CS/PVA blended film.

Entry		Elongation at break /%	Tensile strength (MPa)
1	CS	1.21 ± 0.35	22.33 ± 4.33
2	PVA	12.90 ± 1.44	75.87 ± 2.56
3	CS:PVA = (1:1)	10.07 ± 1.53	62.92 ± 6.28
4	CS:PVA:MMT = (1:1:0.1)	8.70 ± 1.23	50.12 ± 7.87
5	CS:PVA:MMT = (1:1:0.2)	7.20 ± 0.85	42.55 ± 6.63
6	CS:PVA:MMT = (1:1:0.3)	5.26 ± 1.10	38.54 ± 3.70
7	CS:PVA:MMT = (1:1:0.4)	3.02 ± 1.11	30.01 ± 8.64
87	CS:PVA:MMT = (1:1:0.5)	2.67 ± 1.24	25.92 ± 3.28

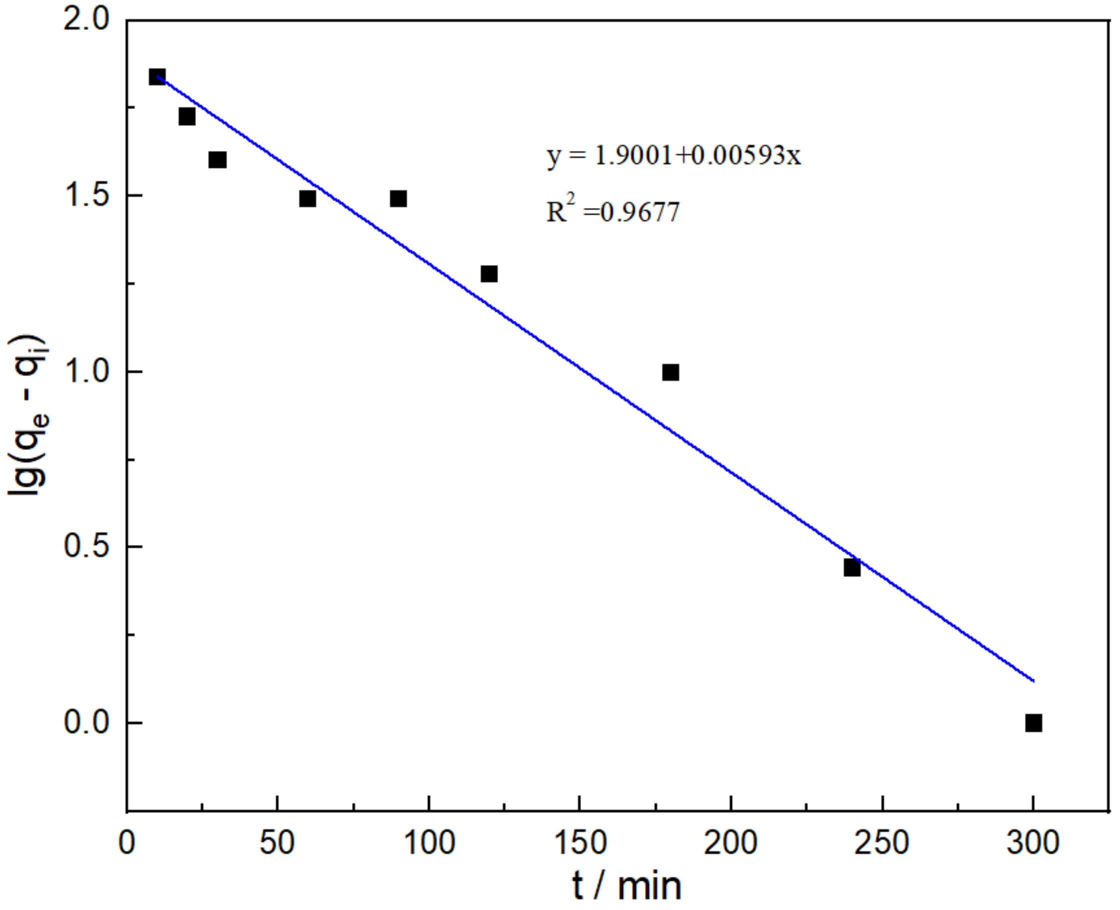

Figure S1. Adsorption of Pd^{2+} by CS/PVA/MMT is consistent with the pseudo–first–order kinetic.

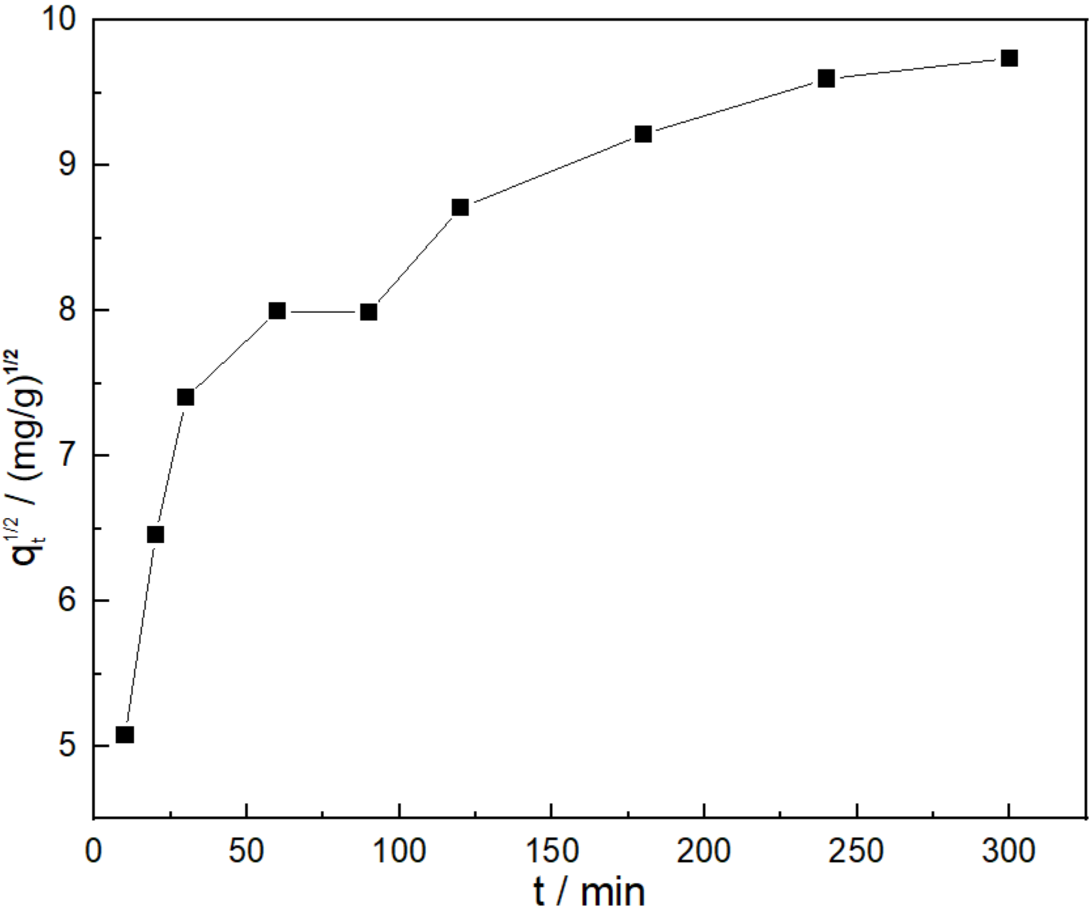

Figure S2. Adsorption of Pd^{2+} by CS/PVA/MMT is consistent with the Weber Morris mode.

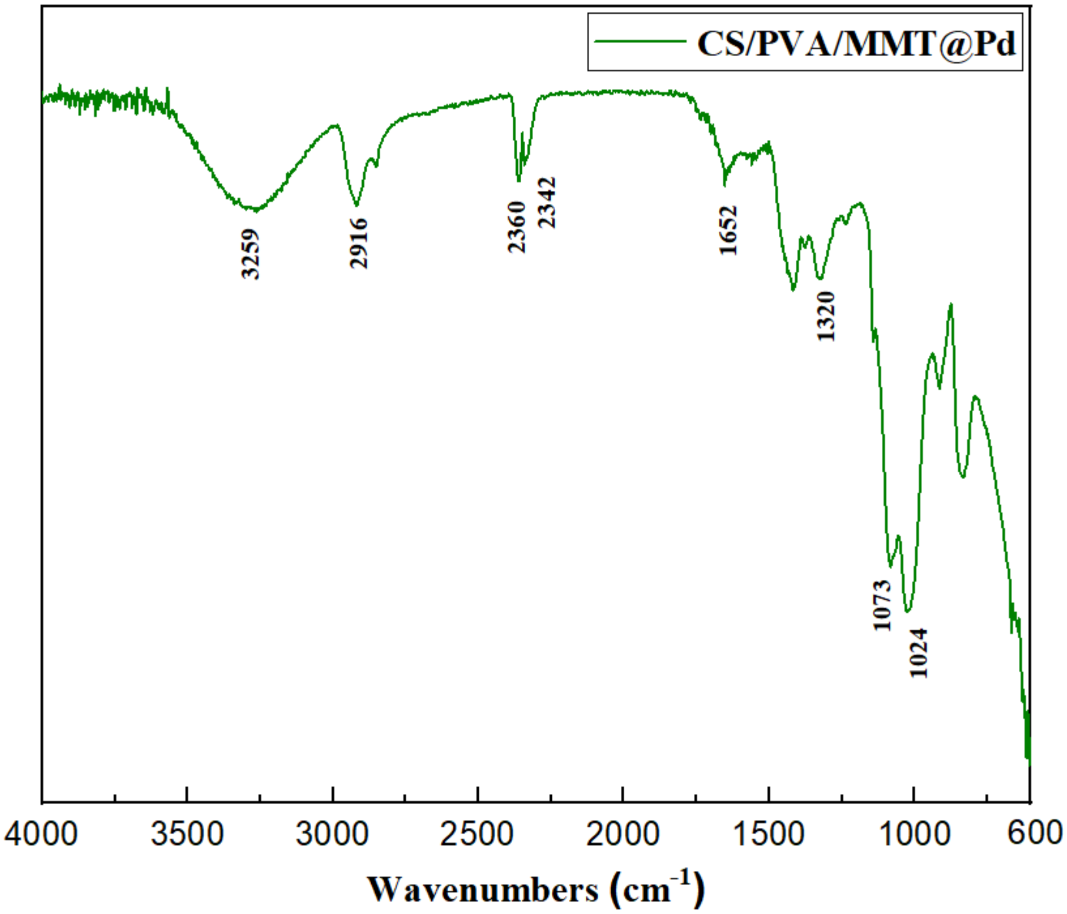

Figure S3. FTIR spectra of CS/PVA/MMT @Pd.

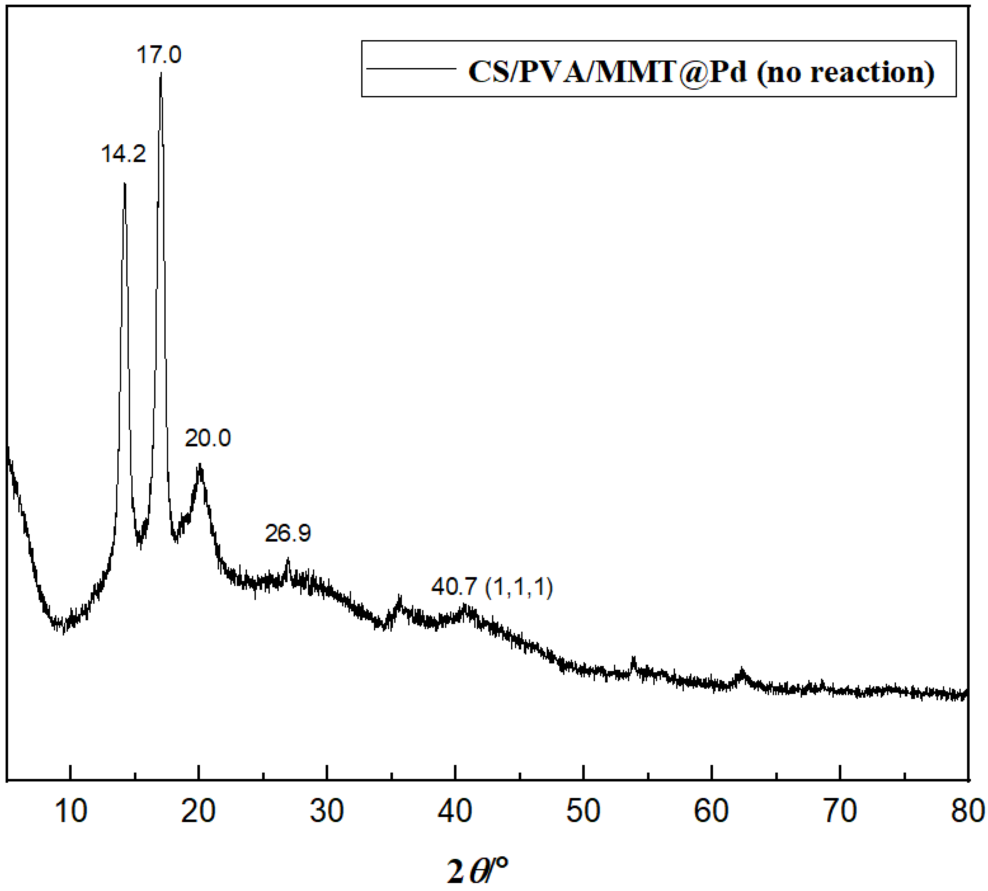

Figure S4. X–ray diffraction patterns of CS/PVA/MMT @Pd (after one reaction).

3. Characterization data for products

3a 3,4'–dimethoxy–1,1'–biphenyl

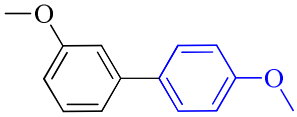

^{1}H NMR (400 MHz, $CDCl_3$, ppm) δ = 7.57–7.54(m, 2H), 7.38–7.34(t, *J* = 8.0 Hz, 2H), 7.18–7.16(d, *J* = 7.6 Hz, 1H), 7.12–7.11(t, *J* = 2.0 Hz, 1H),7.01–6.99(m, 2H), 6.90–6.87(dd, *J* = 8.2 Hz, 2.6 Hz, 1H), 3.88(s, 3H)., 3.87(s, 3H).
^{13}C NMR (100 MHz, $CDCl_3$, ppm) δ = 159.84, 159.16, 142.27, 133.51, 129.66, 128.13, 119.21, 114.09, 112.44, 111.93, 55.28, 55.21.

3b 4'–methoxy–2–methyl–1,1'–biphenyl

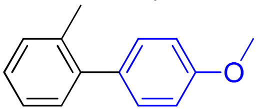

^{1}H NMR (400 MHz, $CDCl_{33}$, ppm) δ = 7.18–7.13(m, 6H), 6.87–6.85(d, *J* = 8.8 Hz, 1H), 3.76(s, 3H), 2.19(s, 3H).
^{13}C NMR (100 MHz, $CDCl_3$, ppm) δ = 158.42, 141.46, 135.42, 134.28, 130.25, 130.20, 129.85, 126.92, 125.71, 113.41, 55.22, 20.54.

3c 4'–methoxy–3–methyl–1,1'–biphenyl

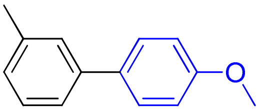

^{1}H NMR (400 MHz, $CDCl_3$, ppm) δ = 7.55–7.51(m, 2H), 7.38–7.36(d, *J* = 9.0 Hz, 2H), 7.43–7.30(t, *J* = 7.2 Hz, 1H), 7.14–7.13 d, *J* = 7.2Hz, 1H), 7.00–6.96(m, 2H), 3.86(s, 3H), 2.42(s, 3H).
^{13}C NMR (101 MHz, $CDCl_3$, ppm) δ = 158.99, 140.75, 138.25, 133.83, 128.60, 128.13, 127.53, 127.38, 123.81, 114.08, 55.31, 21.55.

3d 4–methoxy–4'–methyl–1,1'–biphenyl

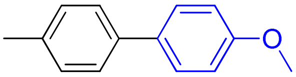

^{1}H NMR (400 MHz, $CDCl_3$, ppm) δ = 7.44–7.40(m, 2H), 7.37–7.35(m, 2H), 7.15–7.12(d, *J*=8.0 Hz, 2H), 6.89–6.86(m, 2H), 3.75(s, 3H), 2.29(s, 3H).
^{13}C NMR (100 MHz, $CDCl_3$, ppm) δ = 158.89, 137.92, 136.28, 133.69, 129.40, 127.90, 126.53, 114.12, 55.27, 21.01.

3e 4'–methoxy–2,6–dimethyl–1,1'–biphenyl

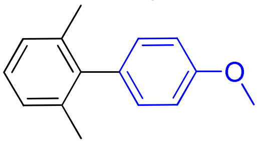

^{1}H NMR (400 MHz, $CDCl_3$, ppm) δ = 7.18–7.15(m, 1H), 7.12–7.07(m, 4H), 6.99–6.97(m, 4H), 3.87(s, 3H), 2.06(s, 3H).
^{13}C NMR (100 MHz, $CDCl_3$, ppm) δ = 158.17, 141.46, 136.50, 133.24, 130.02, 127.20, 126.85, 113.74, 55.18, 20.91.

3f 4'–methoxy–[1,1'–biphenyl]–4–carbonitrile

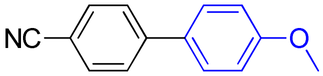

^{1}H NMR (400 MHz, $CDCl_3$, ppm) δ = 7.69–7.62(m, 4H), 7.55–7.52(m, 2H), 7.02–6.99(m, 2H), 3.86(s, 3H).
^{13}C NMR (100 MHz, $CDCl_3$, ppm) δ = 160.09, 145.10, 132.48, 131.36, 128.27, 127.00, 119.04, 114.45, 109.96, 55.32.

3g 4'–methoxy–[1,1'–biphenyl]–4–carbaldehyde

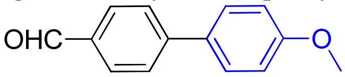

^{1}H NMR (400 MHz, $CDCl_3$, ppm) δ = 10.03(s, 1H), 7.94–7.91(dt, *J* = 8.4 Hz, 1.8 Hz, 2H), 7.73–7.70(dt, *J* = 8.4 Hz, 1.8 Hz, 2H), 7.62–7.58(m, 2H), 7.03–7.00(m, 2H), 3.87(s, 3H).
^{13}C NMR (100 MHz, $CDCl_3$, ppm) δ = 191.89, 160.07, 146.76, 134.63, 132.03, 130.30, 128.48, 127.03, 114.45, 55.38.

3h 4–chloro–4'–methoxy–1,1'–biphenyl

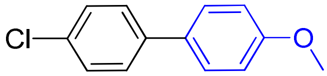

^{1}H NMR (400 MHz, $CDCl_3$, ppm) δ = 7.50–7.46(m, 4H), 7.40–7.36(m, 2H), 7.62–7.58(m, 2H), 3.85(s, 3H).
^{13}C NMR (100MHz, $CDCl_3$, ppm) δ = 159.33, 139.24, 132.64, 132.47, 128.81, 127.99, 127.91, 114.28, 55.35.

3i methyl 4'–methoxy–[1,1'–biphenyl]–4–carboxylate

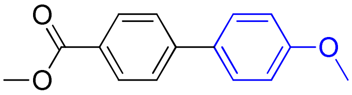

^{1}H NMR (400 MHz, $CDCl_3$, ppm) δ = 8.09–8.06 (m, 2H), 7.64–7.56(m, 4H), 7.01–6.98 (m, 2H), 3.93(s, 3H), 3.85(s, 3H).
^{13}C NMR (100 MHz, $CDCl_3$, ppm) δ = 167.07, 159.76, 145.16, 132.34, 130.07, 128.33, 128.15, 126.43, 114.32, 55.35, 52.09.

3j (4'–methoxy–[1,1'–biphenyl]–4–yl)methanol

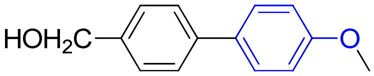

^{1}H NMR (400 MHz, $CDCl_3$, ppm) δ = 7.57–7.51 (m, 4H), 7.43–7.41(d, *J* = 8.2 Hz, 2.0 Hz, 1H), 6.87–6.83 (m, 2H), 6.72–7.70(d, *J* = 8.2 Hz, 1H), 3.98(s, 2H), 3.74(s, 3H).
^{13}C NMR (100 MHz, $CDCl_3$, ppm) δ = 159.13, 140.23, 139.18, 133.30, 128.07, 127.48, 126.86, 114.18, 65.15, 55.34.

3k 3–chloro–3'–methoxy–[1,1'–biphenyl]–4–amine

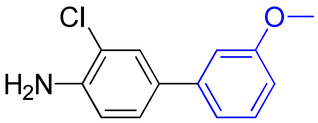

^{1}H NMR (400 MHz, CDCl$_3$, ppm) δ = 7.37–7.32 (m, 2H), 7.19–7.16(dd, *J* = 8.2 Hz, 2H), 7.00–6.96 (m, 2H), 4.73(s, 2H), 3.85(s, 3H).
^{13}C NMR (100 MHz, CDCl$_3$, ppm) δ = 158.64, 141.55, 132.54, 132.11, 127.37, 127.35, 125.87, 119.61, 116.08, 114.13, 55.29.

3l 4–chloro–2–(4–methoxycyclohexa–2,4–dien–1–yl)pyrimidine

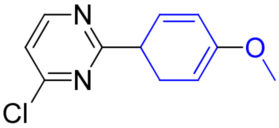

^{1}H NMR (400 MHz, CDCl$_3$, ppm) δ = 8.57–8.56 (d, *J* = 5.2 Hz, 1H), 8.09–8.06(d, *J* = 8.8 Hz, 2H), 7.57–7.56 (d, *J* = 5.2 Hz, 1H), 7.02–7.00 (d, *J* = 8.8 Hz, 2H), 3.89(s, 3H).
^{13}C NMR (100 MHz, CDCl$_3$, ppm) δ = 166.61, 162.76, 159.41, 129.13, 127.34, 114.43, 114.17, 55.48.

3m 2–(4–methoxycyclohexa–2,4–dien–1–yl)–4–methylpyrimidine

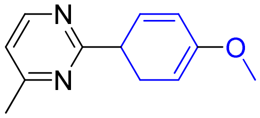

^{1}H NMR (400 MHz, CDCl$_3$, ppm) δ = 8.60–8.59 (d, *J* = 5.2 Hz, 1H), 8.41–8.37(m, 2H), 7.01–6–7.98 (m, 3H), 3.88(s, 3H), 2.57(s, 3H).
^{13}C NMR (100 MHz, CDCl$_3$, ppm) δ = 167.18, 164.02, 161.67, 156.61, 130.36, 129.73, 117.89, 113.81, 99.93, 55.35, 24.45.

3n 3–(4–methoxyphenyl)quinoline

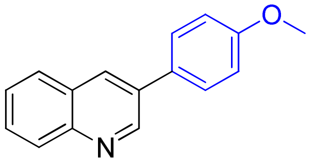

^{1}H NMR (400 MHz, CDCl$_3$, ppm) δ = 9.20–9.19(d, *J* = 2.4 Hz, 1H), 8.36– 8.36 (d, *J* = 2.4 Hz, 1H), 8.35, 8.16–8.14(d, *J* = 8F.4 Hz, 1H), 8.12–8.09(m, 2H), 7.91–7.89(m, 1H), 7.83–7.80(m, 2H), 7.78–7.74(m, 1H), 7.62–7.58(m, 1H), 2.67(s, 3H).
^{13}C NMR (100 MHz, CDCl$_3$, ppm) δ = 197.62, 159.73, 149.75, 146.86, 144.27, 136.47, 133.43, 132.41, 130.18, 129.08, 129.04, 128.97, 128.47, 128.08, 127.83, 127.40, 126.93, 114.61, 55.38, 26.70.

3o 3–(4–methoxyphenyl)thiophene

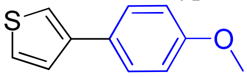

^{1}H NMR (400 MHz, CDCl$_3$, ppm) δ = 7.55–7.51(m, 2H) 7.38–7.34(m, 3H), 6.96–6.92(m, 2H), 3.84(s, 3H)
^{13}C NMR (100 MHz, CDCl$_3$, ppm) δ = 158.8, 141.99, 128.72, 127.57, 126.26, 126.10, 118.95, 114.17, 55.36.

3p 5–(4–methoxyphenyl)thiophene–2–carbaldehyde

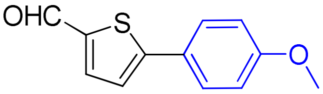

^{1}H NMR (400 MHz, CDCl$_3$, ppm) δ = 9.85 (s, 1H), 7.72–7.71(m, 1H), 7.62–7.60 (dd, *J* = 6.6 Hz, 2.2 Hz, 2H), 7.30–7.29 (d, *J* = 4.0Hz, 1H), 6.96–6.94(dd, *J* = 6.6 Hz, 2.2 Hz, 2H), 3.85(s, 3H)
^{13}C NMR (100 MHz, CDCl$_3$, ppm) δ = 182.74, 160.61, 154.50, 141.36, 137.83, 127.76, 125.66, 122.95, 114.50, 55.40.

3q 3–methoxy–1,1'–biphenyl

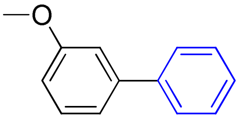

^{1}H NMR (400 MHz, $CDCl_3$, ppm) δ = 7.62–7.60 (d, J = 8.2Hz, 2H), 7.48–7.44 (t, J = 7.2 Hz, 2H), 7.40–7.35 (m, 2H), 7.22–7.20(dd, J = 7.6 Hz, 1.0 Hz, 1H), 7.16–7.15 (m, 1H), 6.93–6.91(m, 1H),3.88(s, 3H)
^{13}C NMR (100 MHz, $CDCl_3$, ppm) δ = 159.84, 142.71, 141.03, 129.72, 128.70, 127.38, 127.16, 119.64, 112.82, 112.60, 55.26.

3r [1,1'–biphenyl]–4–ylmethanol

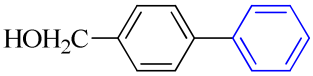

^{1}H NMR (400 MHz, $CDCl_3$, ppm) δ = 7.62–7.60(d, J = 8.0 Hz, 4H), 7.48–7.44(t, J = 7.8 Hz, 4H), 7.38–7.35(t, J = 7.2 Hz, 1H), 4.74(s, 2H).
^{13}C NMR (100 MHz, $CDCl_3$, ppm) δ = 140.75, 140.56, 139.81, 128.74, 127.42, 127.27, 127.04, 65.02.

3s 4–methyl–2–phenylpyrimidine

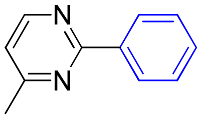

^{1}H NMR (400 MHz, $CDCl_3$, ppm) δ = 8.66– 8.64(d, J = 5.2 Hz, 1H), 8.44–8.42(m, 2H), 7.50–.47(m, 3H), 7.06–7.05(d, J=5.2 Hz, 1H), 2.59(s, 3H).
^{13}C NMR (100 MHz, $CDCl_3$, ppm) δ = 167.27, 164.35, 156.77, 137.78, 130.51, 128.51, 128.12, 118.59, 24.43.

3t 5–phenylthiophene–2–carbaldehyde

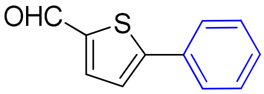

^{1}H NMR (400 MHz, $CDCl_3$, ppm) δ = 9.89(s, 1H), 7.75– 7.74(d, J = 4.0 Hz, 1H), 7.69–7.66(m, 2H), 7.46–7.39(m, 4H).
^{13}C NMR (100 MHz, $CDCl_3$, ppm) δ = 182.80, 154.27, 142.40, 137.41, 132.99, 129.41, 129.17, 126.40, 124.06.

3u 5–phenylthiophene–2–carbaldehyde

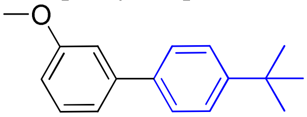

^{1}H NMR (400 MHz, $CDCl_3$, ppm) δ = 7.56– 7.54(m, 2H), 7.50–7.47(m, 2H), 7.38–7.34(t, J = 15.8 Hz, 1H), 7.21–7.14(m, 2H), 6.91–6.88(m, 1H), 3.87 (s, 3H), 1.38(s, 9H).
^{13}C NMR (100 MHz, $CDCl_3$, ppm) δ = 159.84, 150.40, 142.58, 138.15, 129.65, 126.80, 125.66, 119.54, 112.71, 112.37, 55.25, 34.52, 31.35.

3v (4'–(tert–butyl)–[1,1'–biphenyl]–4–yl)methanol

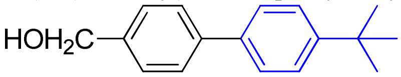

^{1}H NMR (400 MHz, $CDCl_3$, ppm) δ = 7.61– 7.58(m, 2H), 7.56–7.53(dt, J = 8.6 Hz, 2.2 Hz, 2H), 7.49–7.46(dt, J = 8.6 Hz, 2.2 Hz, 2H), 7.45–7.42(dt, J = 8.6 Hz, 2.2 Hz, 2H), 7.38–7.34(t, J = 15.8 Hz, 1H), 4.74 (s, 2H), 1.36(s, 9H).
^{13}C NMR (100 MHz, $CDCl_3$, ppm) δ = 150.33, 140.46, 139.50, 137.85, 127.44, 127.16, 126.69, 125.73, 65.16, 34.52, 31.34.

3w 2–(4–(tert–butyl)phenyl)–4–methylpyrimidine

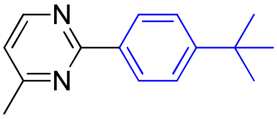

^{1}H NMR (400 MHz, $CDCl_3$, ppm) δ = 8.64– 8.62(d, J = 5.0 Hz, 1H), 8.36– 8.32(m, 2H), 7.52– 7.48(m, 2H), 7.03–7.02(d, J = 5.0 Hz, 1H), 2.58(s, 3H), 1.36(s, 9H).
^{13}C NMR (100 MHz, $CDCl_3$, ppm) δ = 167.18, 164.38, 156.69, 153.80, 135.01, 127.87, 125.49, 118.30, 34.81, 31.23, 24.44.

3x 3'–methoxy–[1,1'–biphenyl]–4–carbonitrile

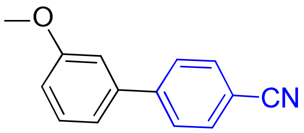

^{1}H NMR (400 MHz, $CDCl_3$, ppm) δ = 7.73–7.66(ddd, *J* = 18.6Hz, 6.6 Hz, 2.2 Hz, 4H), 7.42–7.38 (t, *J* = 8.0 Hz, 1H), 7.18–7.15(dq, *J* = 7.6Hz, 1.0 Hz, 1H), 7.11–7.10 (t, *J* = 2.2 Hz, 1H), 6.98–6.95(ddd, *J* = 8.4Hz, 2.6 Hz, 1.0Hz, 1H), 3.87(s, 3H).
^{13}C NMR (100 MHz, $CDCl_3$, ppm) δ = 160.03, 145.43, 140.54, 132.49, 130.10, 127.71, 119.57, 118.87, 113.79, 113.00, 110.91, 55.32.

3y 4'–(hydroxymethyl)–[1,1'–biphenyl]–4–carbonitrile

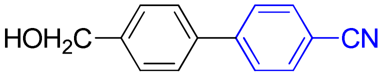

^{1}H NMR (400 MHz, $CDCl_3$, ppm) δ = 7.72–7.66(qd, *J* = 6.2Hz, 1.8 Hz, 4H), 7.60–7.57 (dd, *J* = 6.2 Hz, 1.8 Hz, 2H), 7.49–7.47(d, *J* = 8.2Hz, 2H), 4.76(s, 2H).
^{13}C NMR (100 MHz, $CDCl_3$, ppm) δ = 145.23, 141.41, 138.33, 132.57, 127.58, 127.55, 127.32, 118.91, 110.77, 64.71.

3z 4–(thiophen–3–yl)benzonitrile

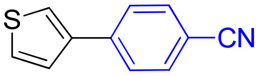

^{1}H NMR (400 MHz, $CDCl_3$, ppm) δ = 7.69(s, 4H), 7.59–7.57 (q, *J* = 1.4 Hz, 1H), 7.46–7.44(dd, *J* = 5.0 Hz , 3.0 Hz, 1H), 7.41–7.40(dd, *J* = 5.0 Hz, 1.4 Hz, 1H).
^{13}C NMR (100 MHz, $CDCl_3$, ppm) δ = 140.30, 139.96, 132.68, 127.14, 126.80, 125.89, 122.59, 118.96, 110.40.

3aa 4–nitro–1,1'–biphenyl

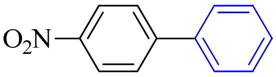

^{1}H NMR (400 MHz, $CDCl_3$, ppm) δ =8.32–8.29(m, 2H), 7.64–7.61(m, 2H), 7.76–7.72(m, 2H), 7.53–7.43(m, 3H).
^{13}C NMR (100 MHz, ppm) δ= 147.61, 147.05, 138.74, 129.13, 128.89, 127.78, 127.36, 124.09.

3ab 4–methoxy–4'–nitro–1,1'–biphenyl

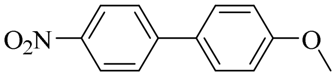

^{1}H NMR (400 MHz, $CDCl_3$, ppm) δ = 8.28–8.25(m, 2H), 7.64–7.61(m, 2H), 7.71–7.67(m, 2H), 7.60–7.56(m, 2H), 7.04–7.00(m, 2H), 3.87(s,3H).
^{13}C NMR (100 MHz, ppm) δ= 160.40, 147.17, 146.49, 131.03, 128.54, 127.04, 124.11, 114.57, 55.40.

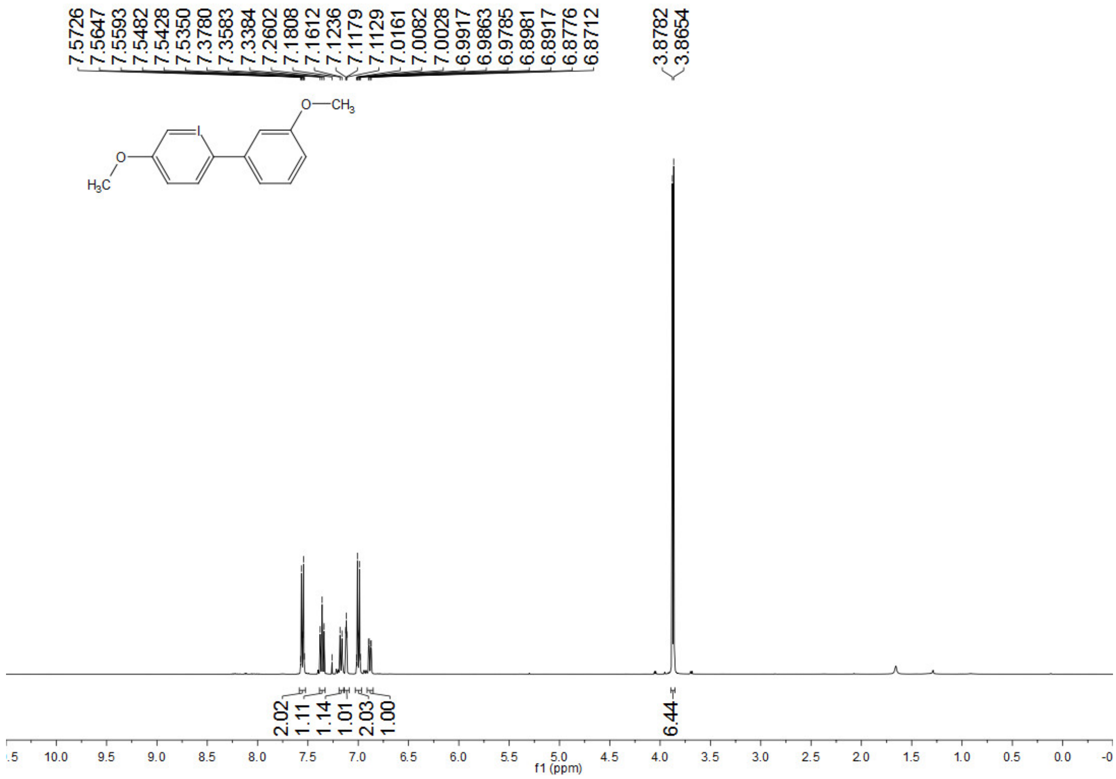

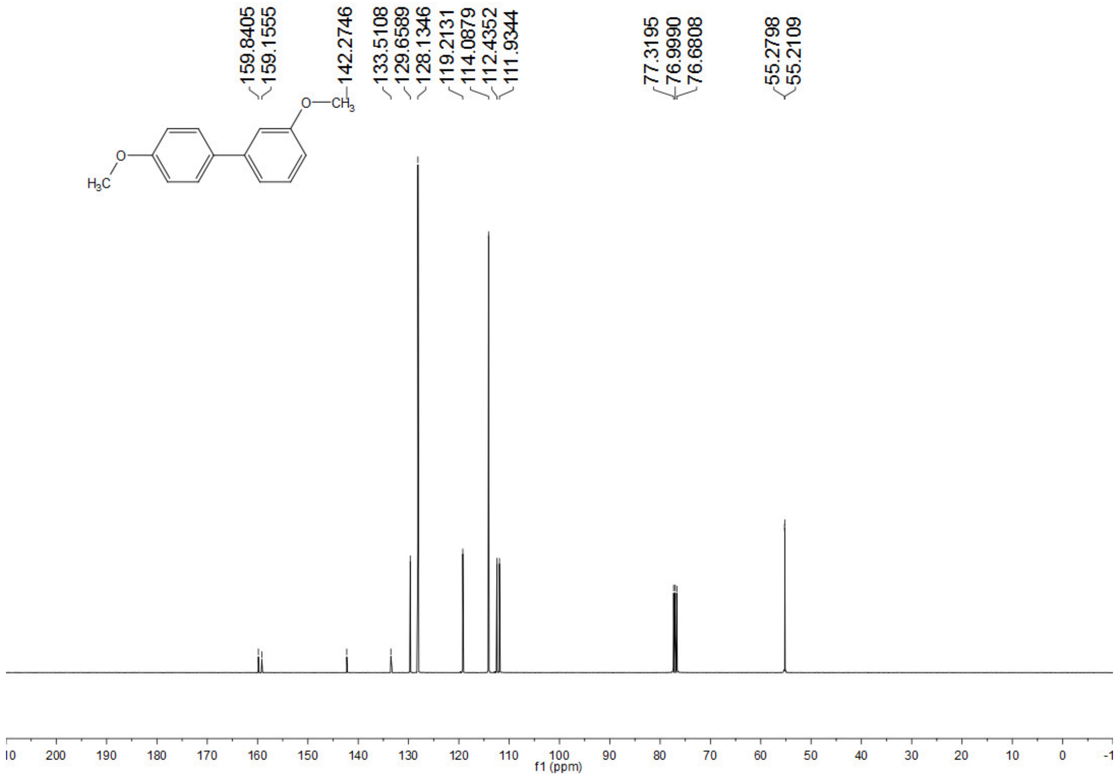

Figure S5. ^{1}H NMR and ^{13}C NMR spectra of **3a.**

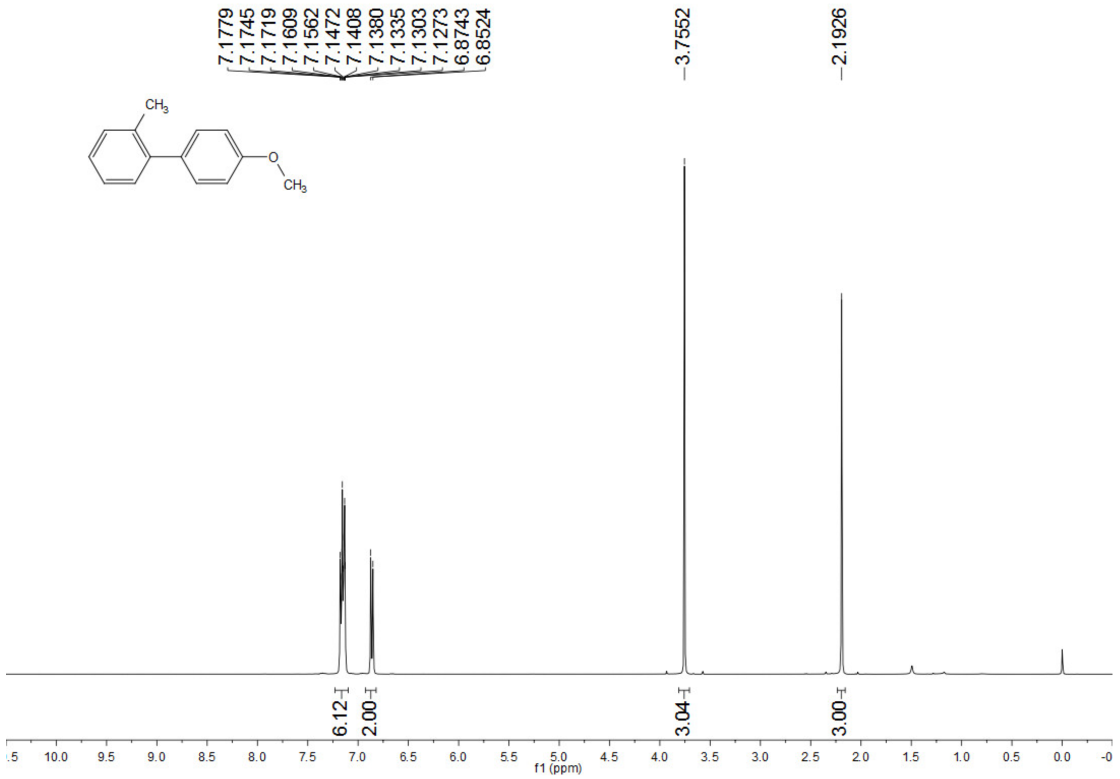

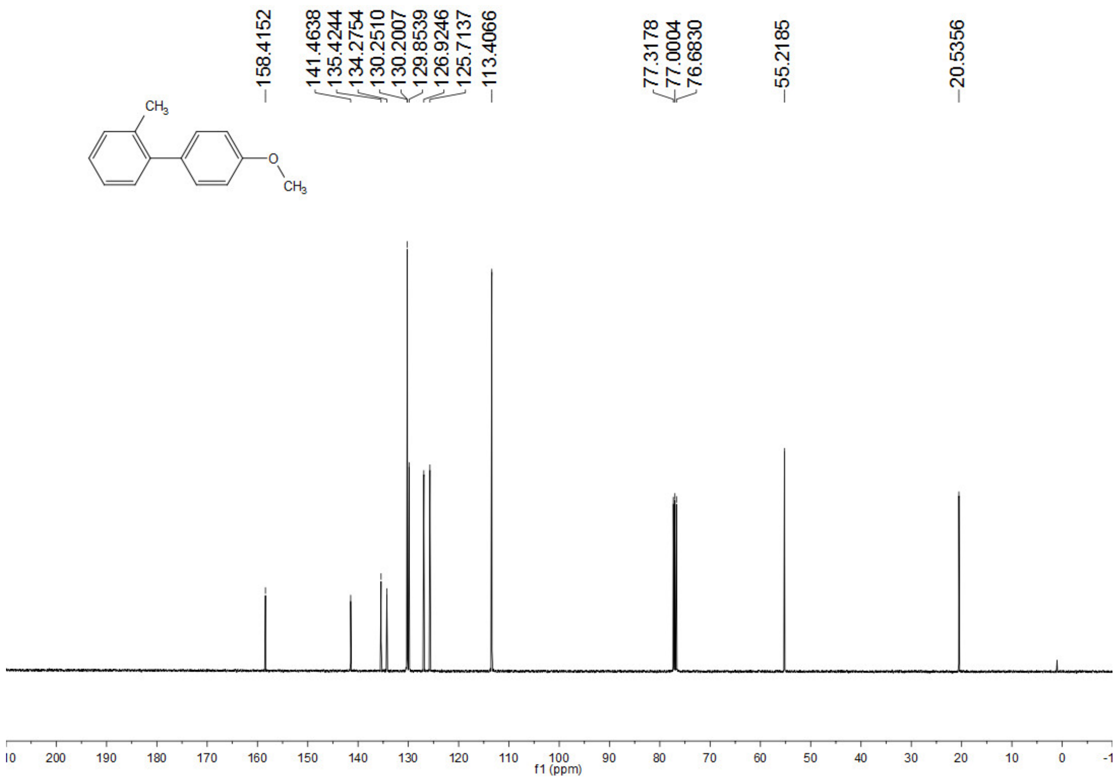

Figure S6. ^{1}H NMR and ^{13}C NMR spectra of **3b.**

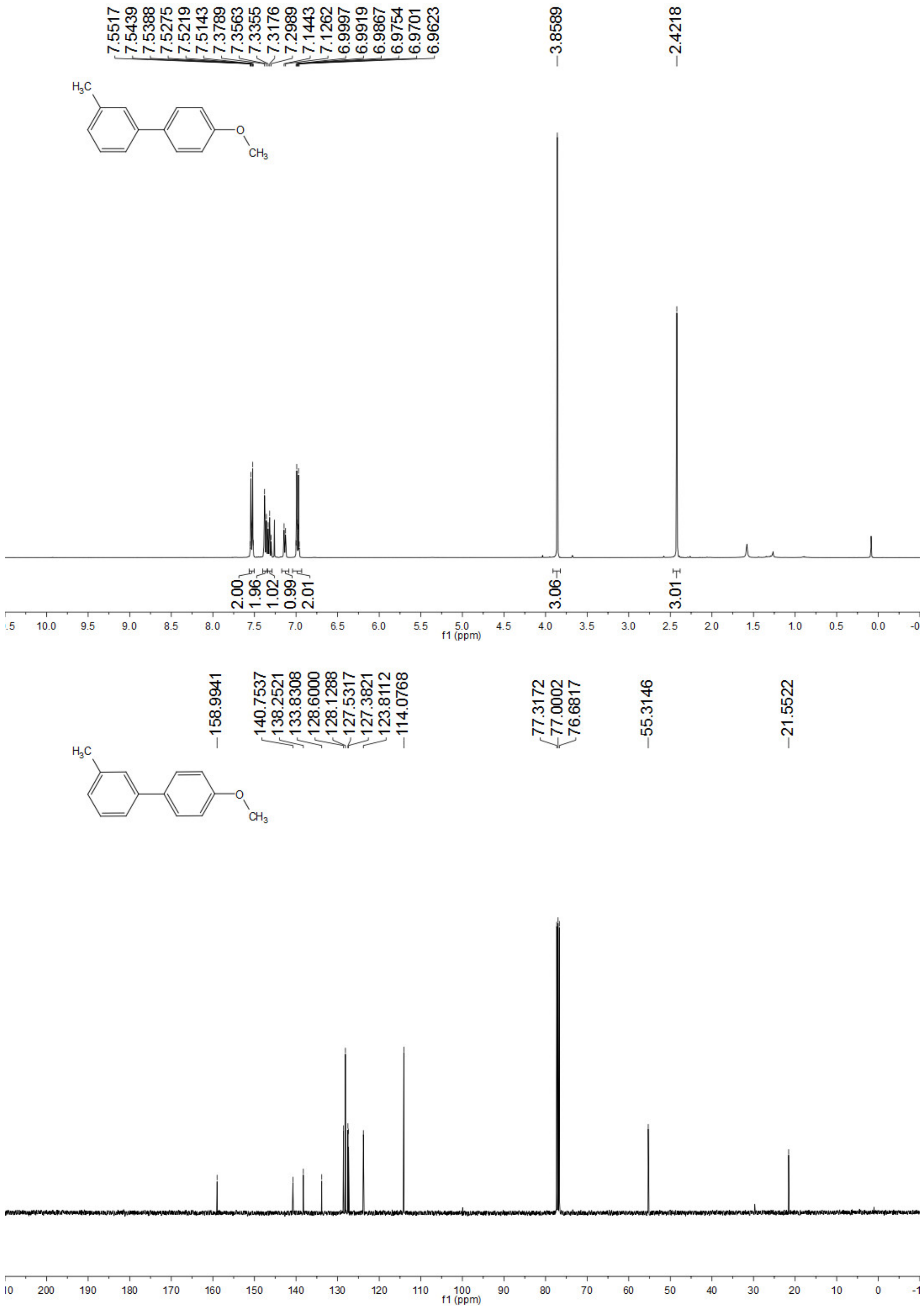

Figure S7. ^{1}H NMR and ^{13}C NMR spectra of **3c.**

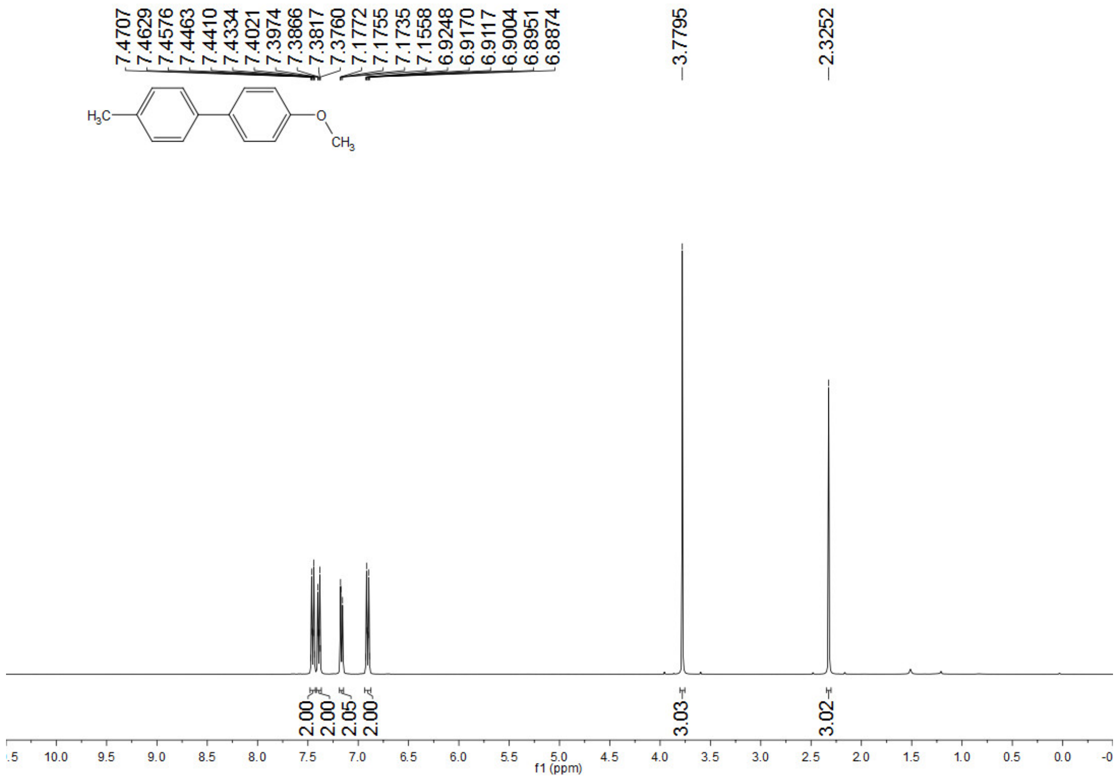

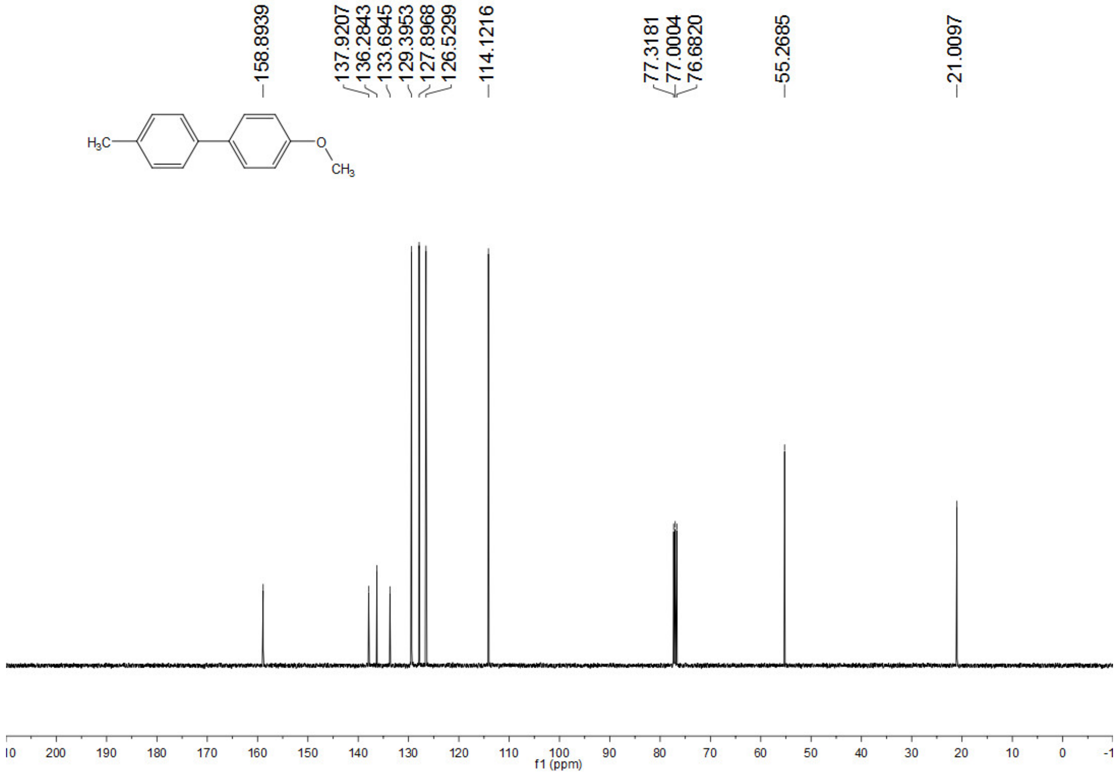

Figure S8. ^{1}H NMR and ^{13}C NMR spectra of **3d.**

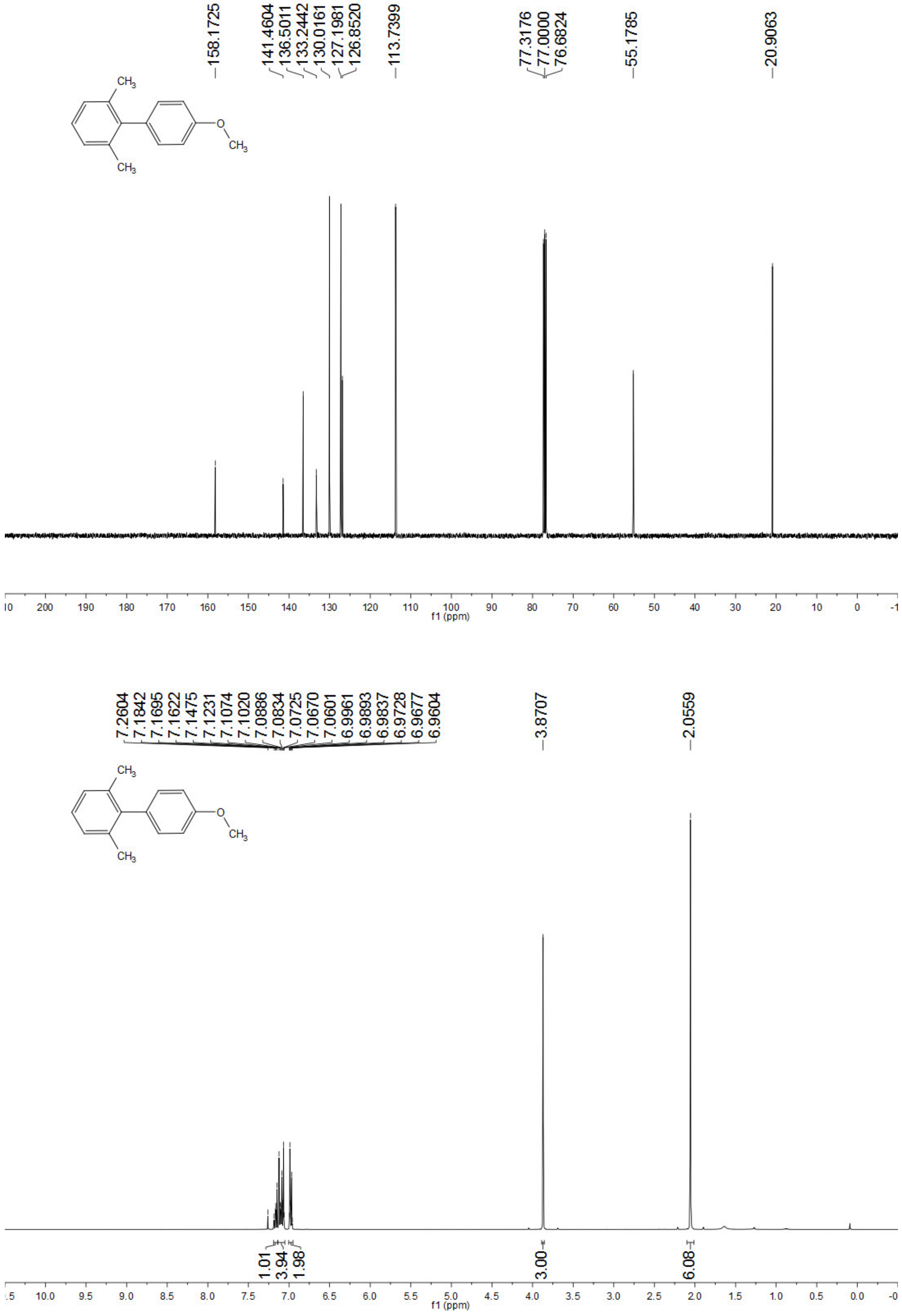

Figure S9. ^{1}H NMR and ^{13}C NMR spectra of **3e.**

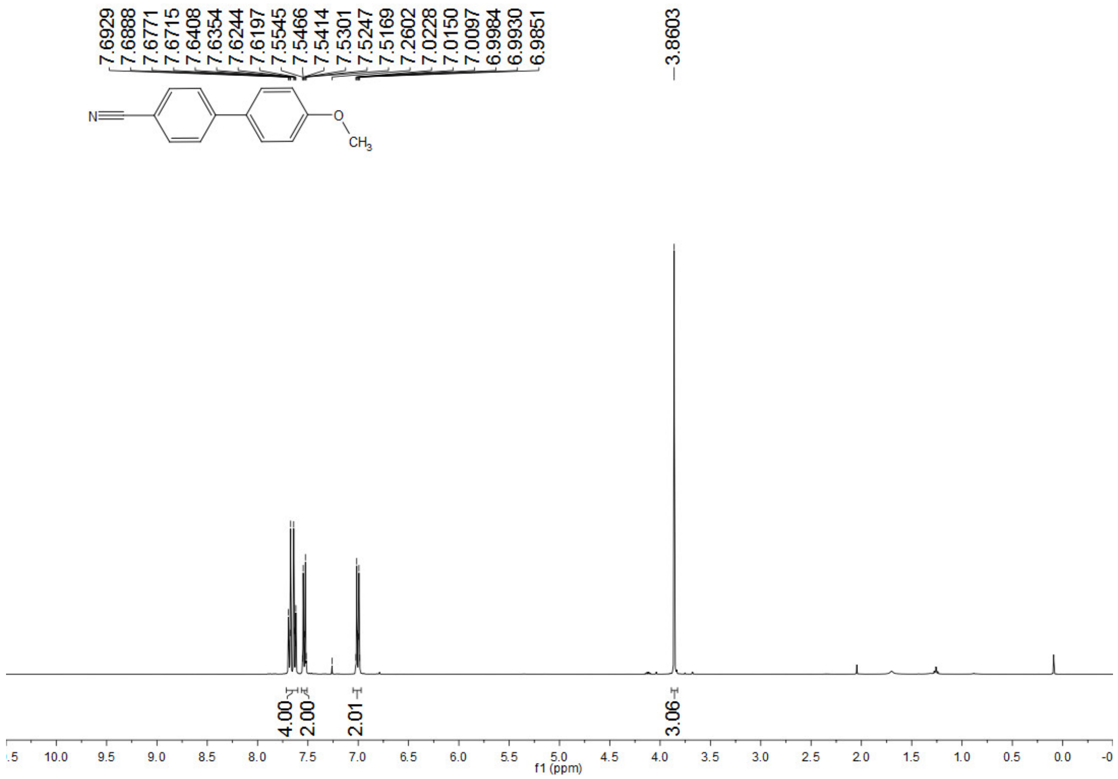

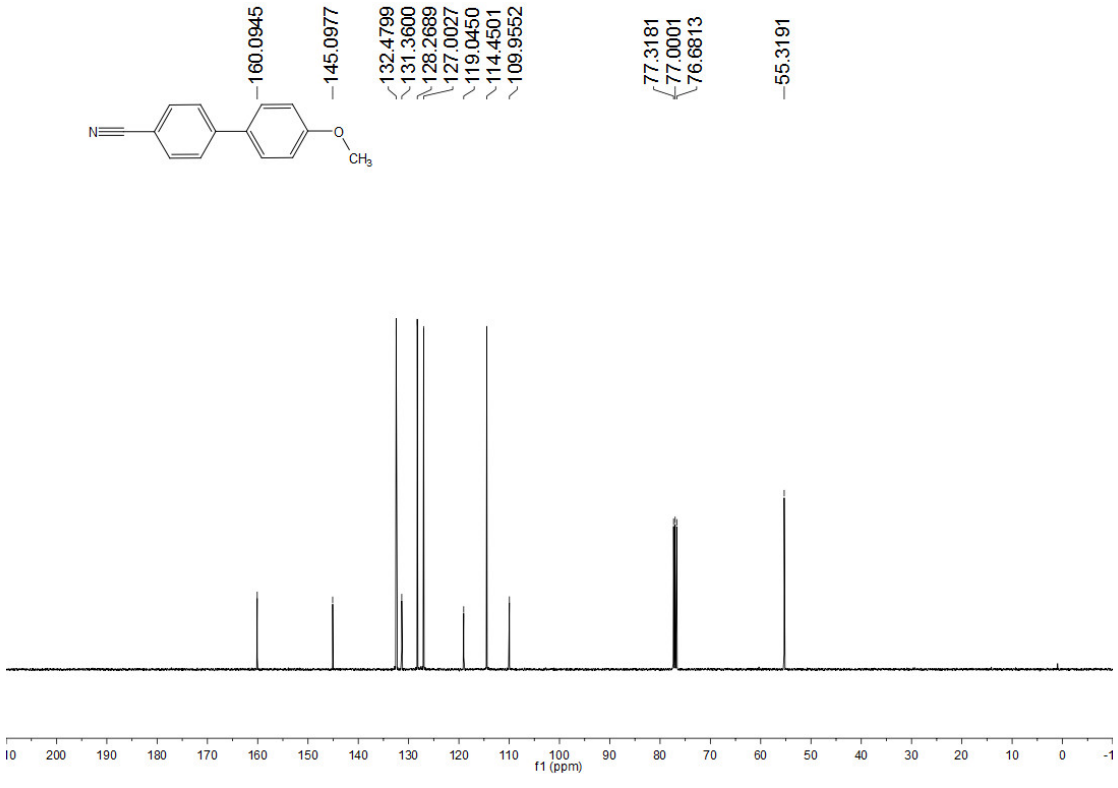

Figure S10. ^1H NMR and ^{13}C NMR spectra of **3f.**

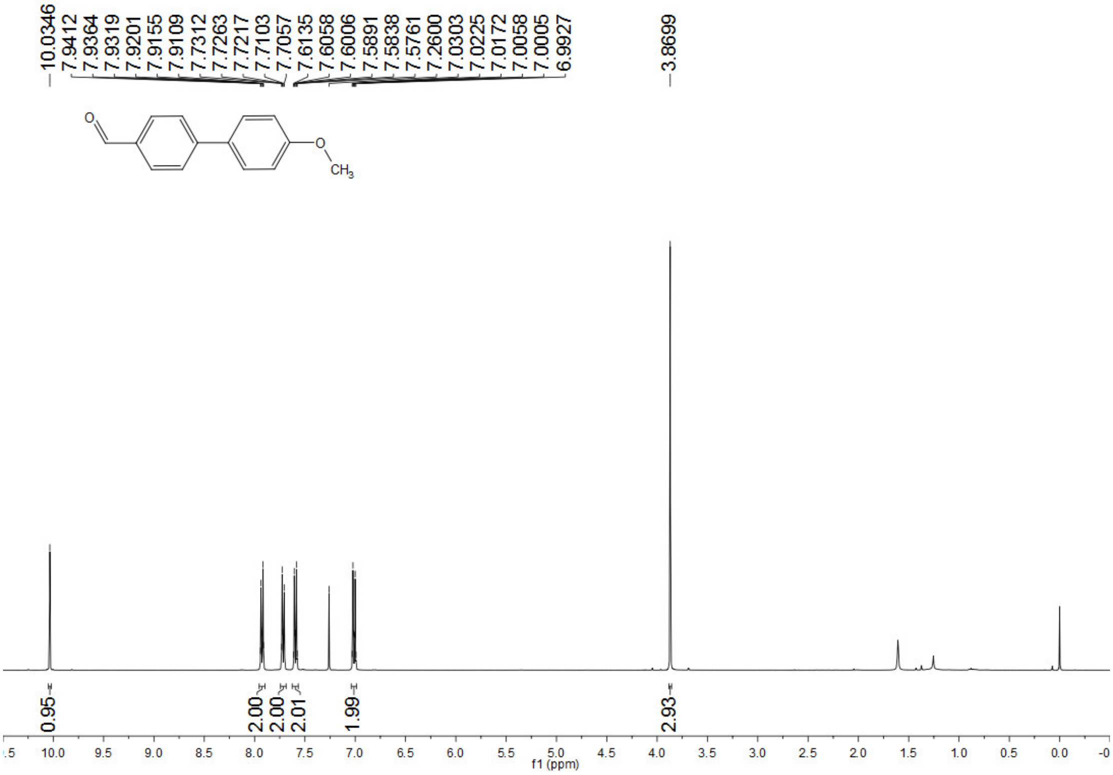

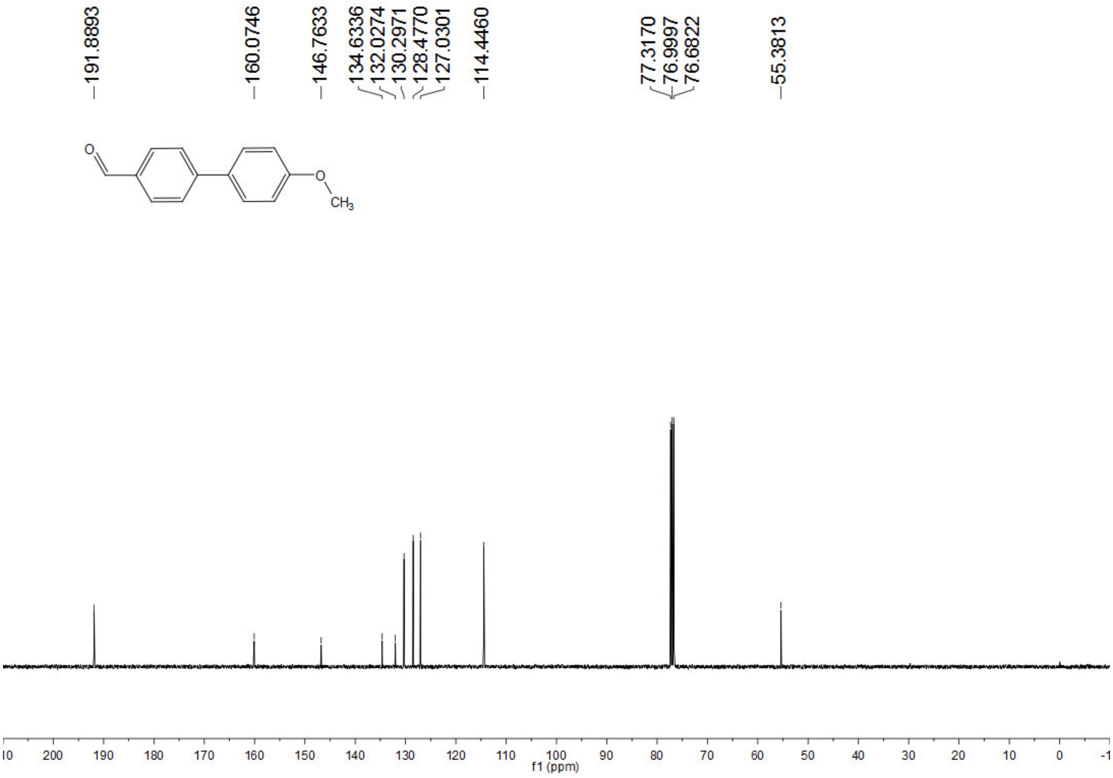

Figure S11. ^{1}H NMR and ^{13}C NMR spectra of **3g.**

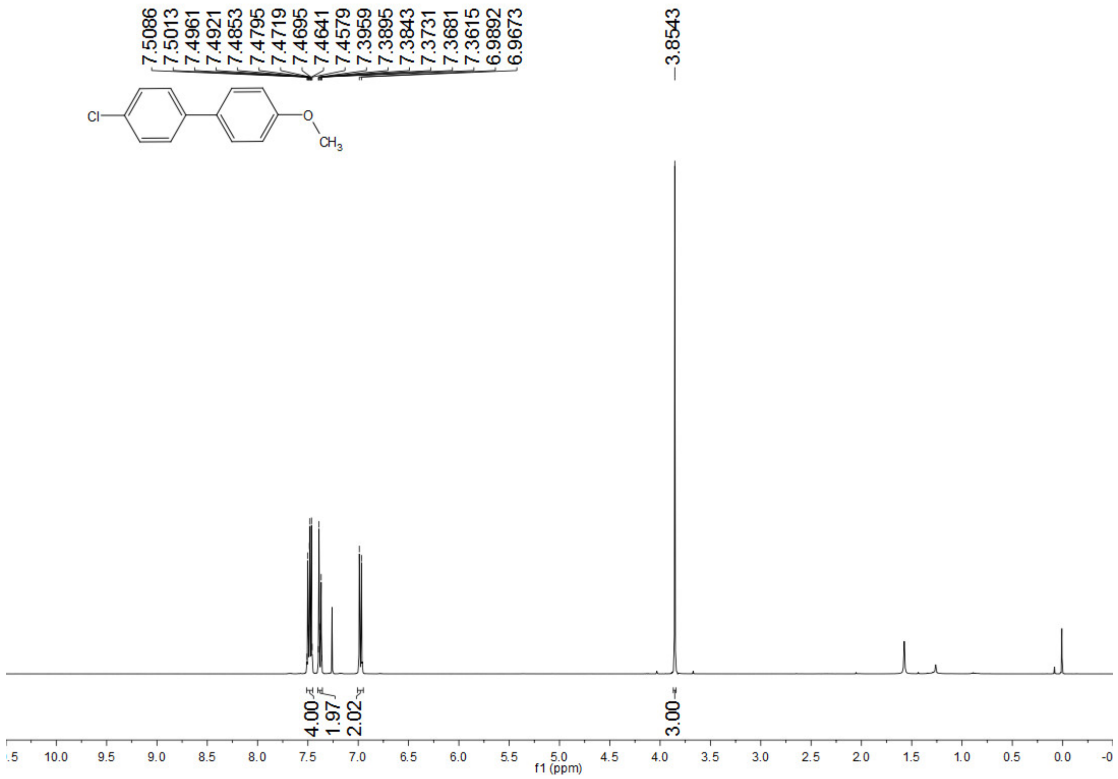

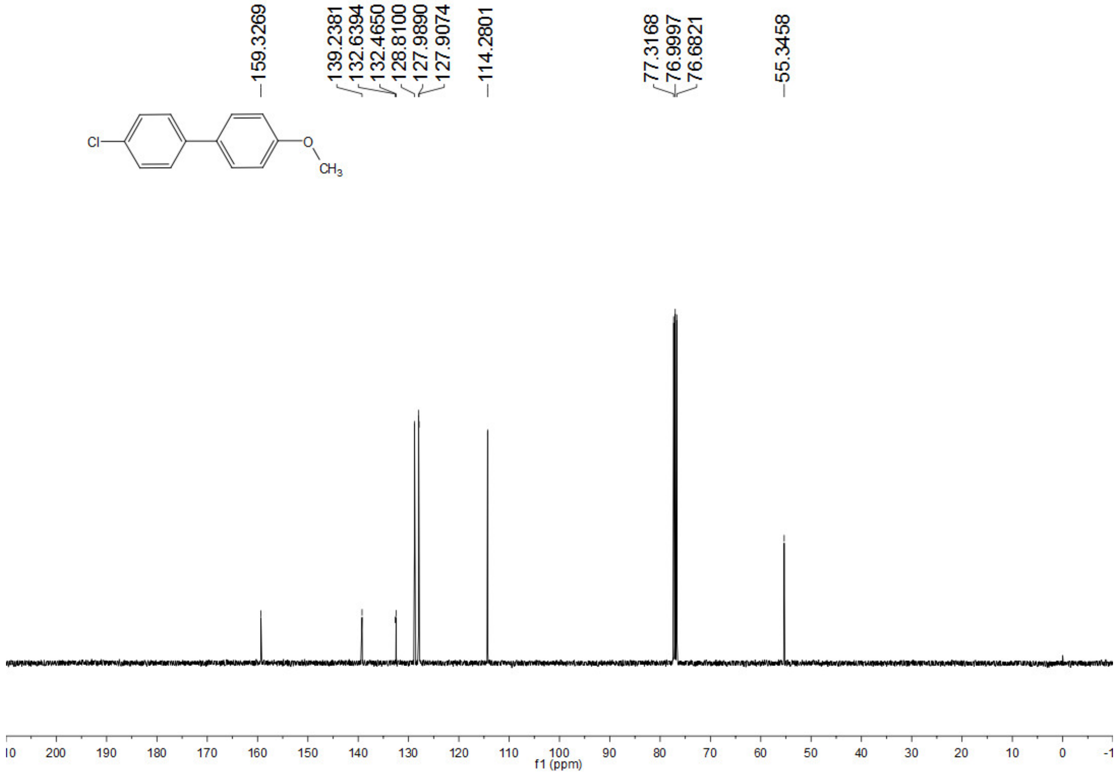

Figure S12. ^{1}H NMR and ^{13}C NMR spectra of **3h.**

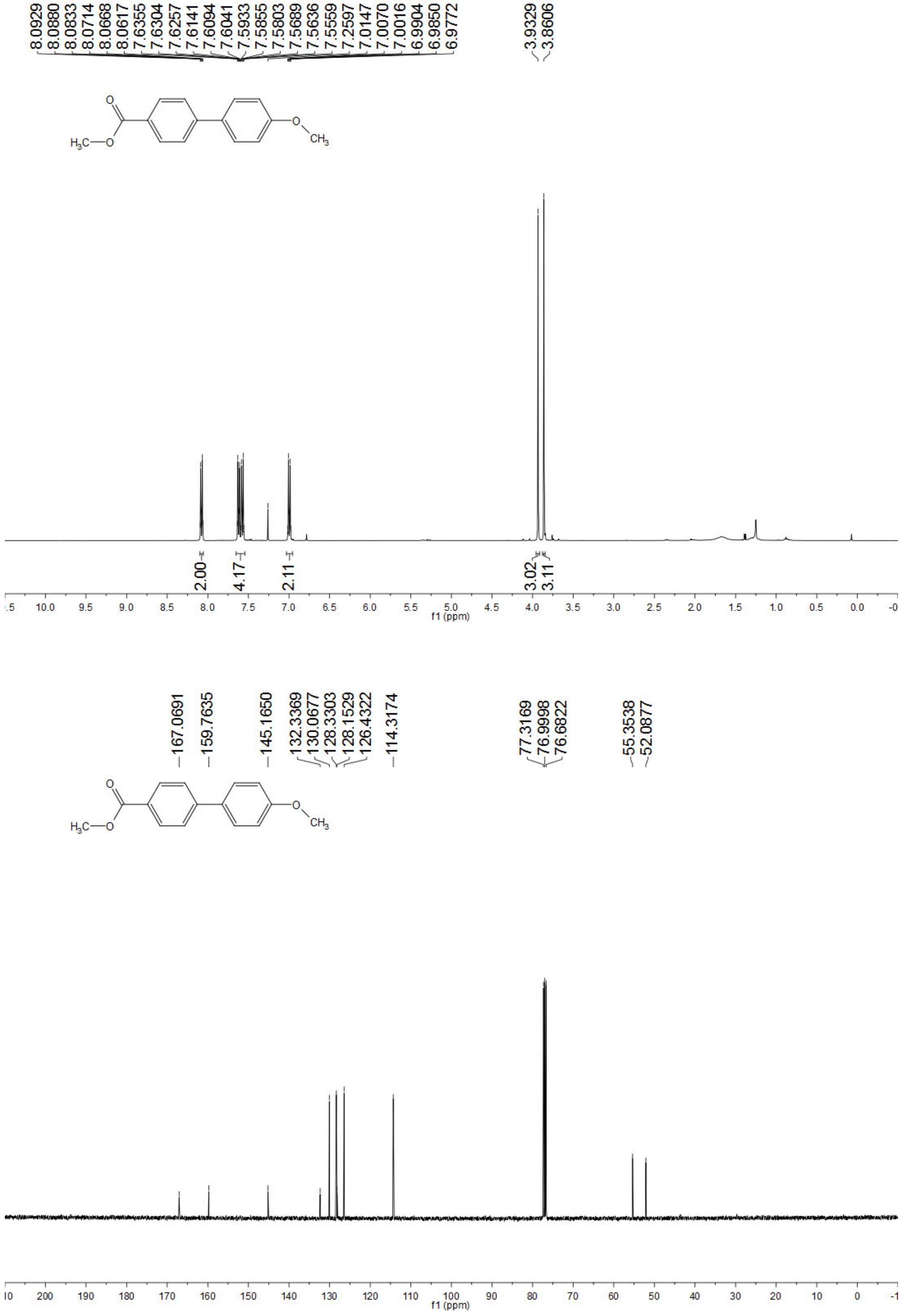

Figure S13. ^{1}H NMR and ^{13}C NMR spectra of **3i.**

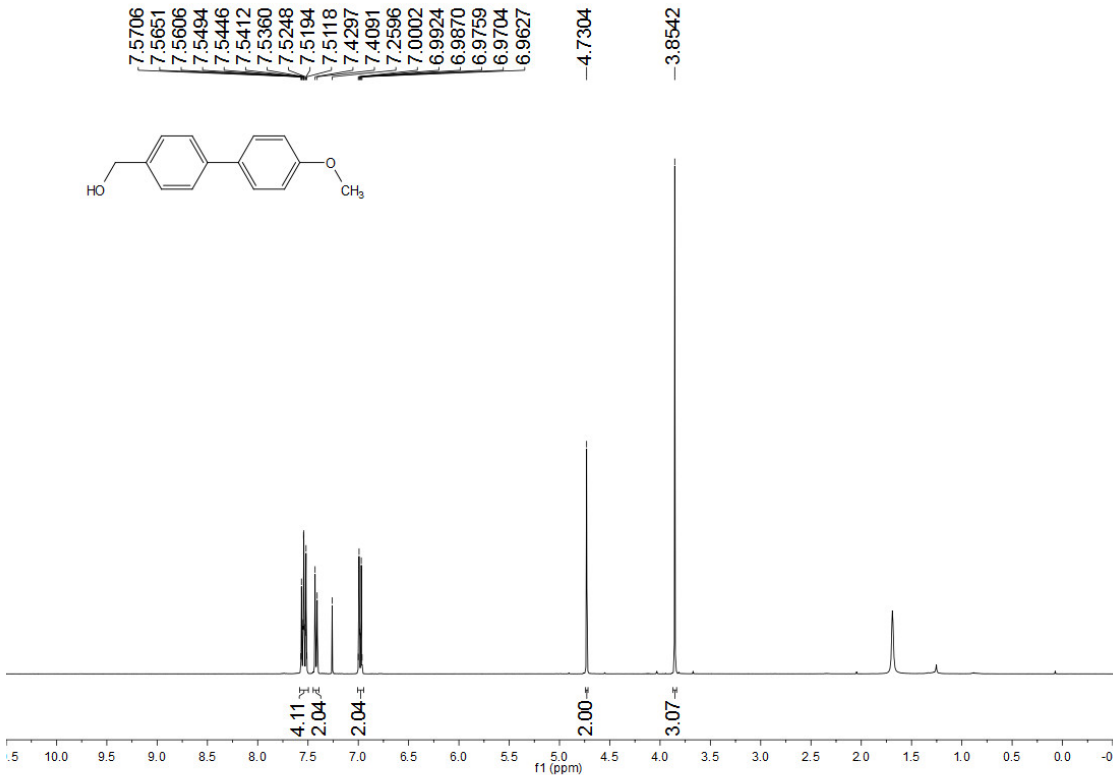

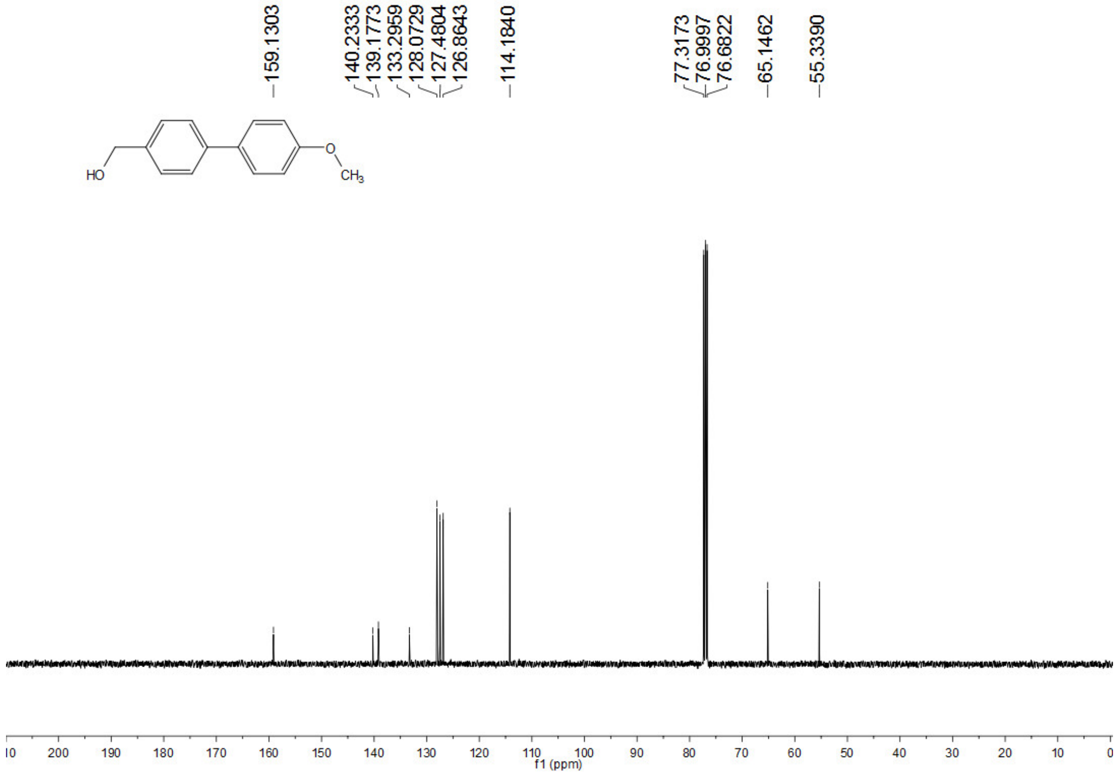

Figure S14. ^{1}H NMR and ^{13}C NMR spectra of **3j.**

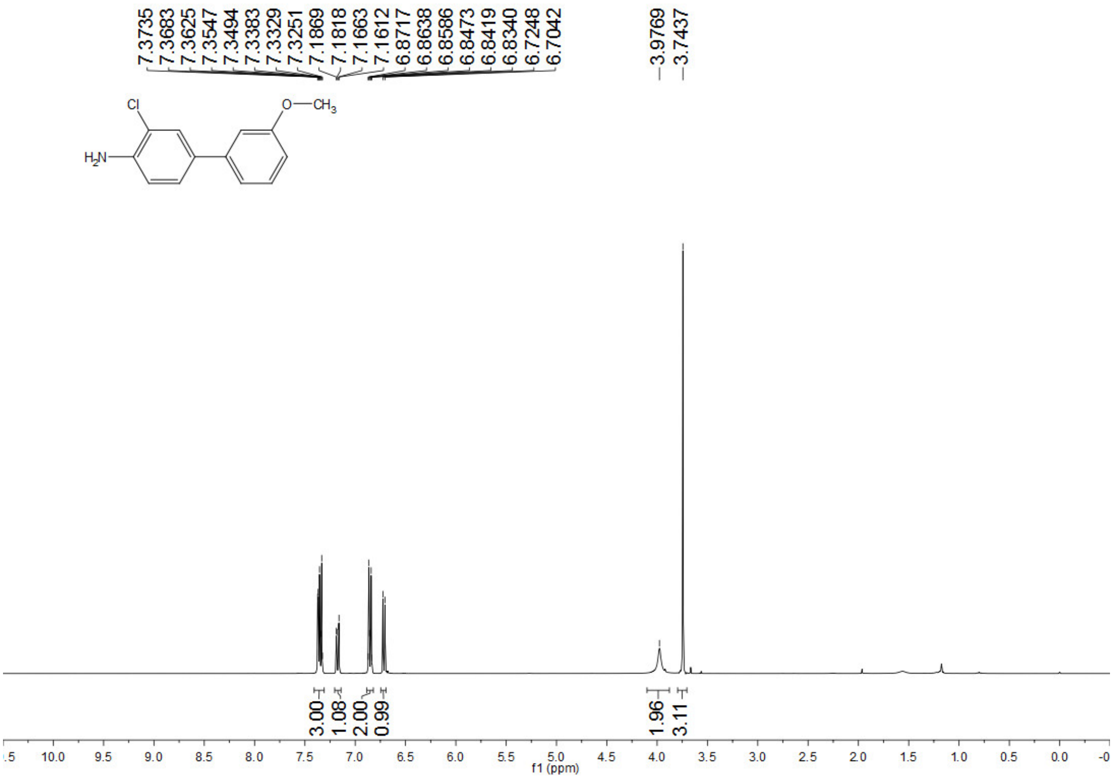

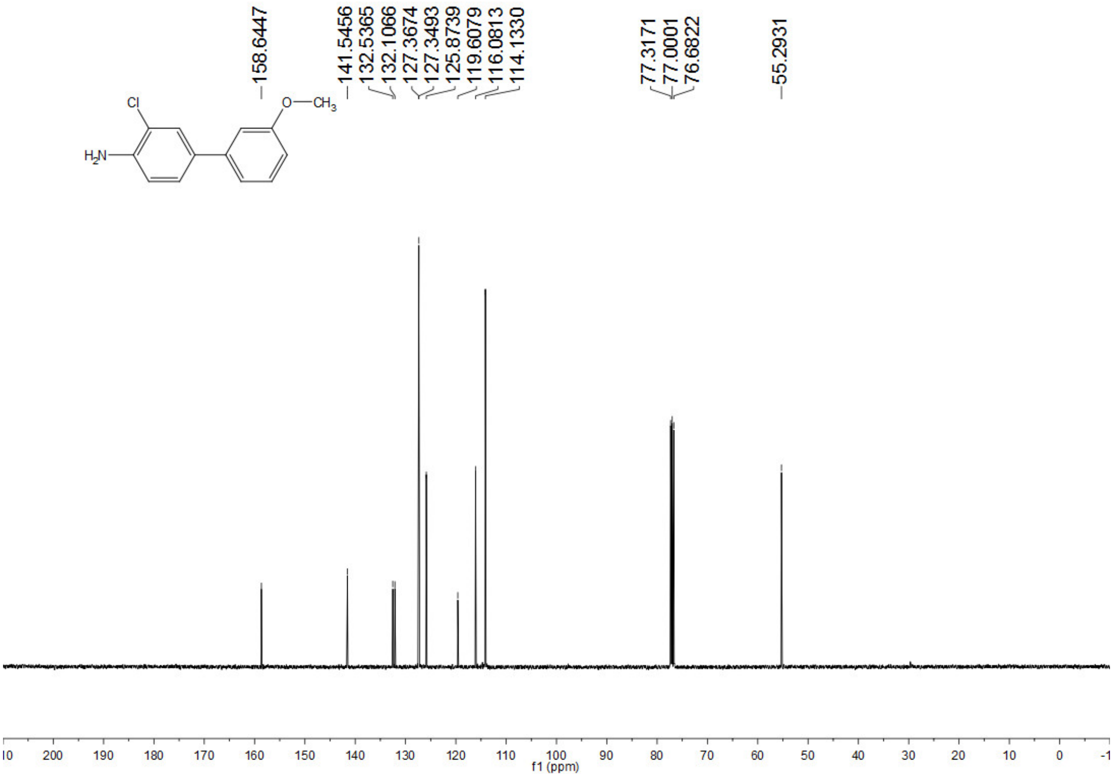

Figure S15. ^{1}H NMR and ^{13}C NMR spectra of **3k.**

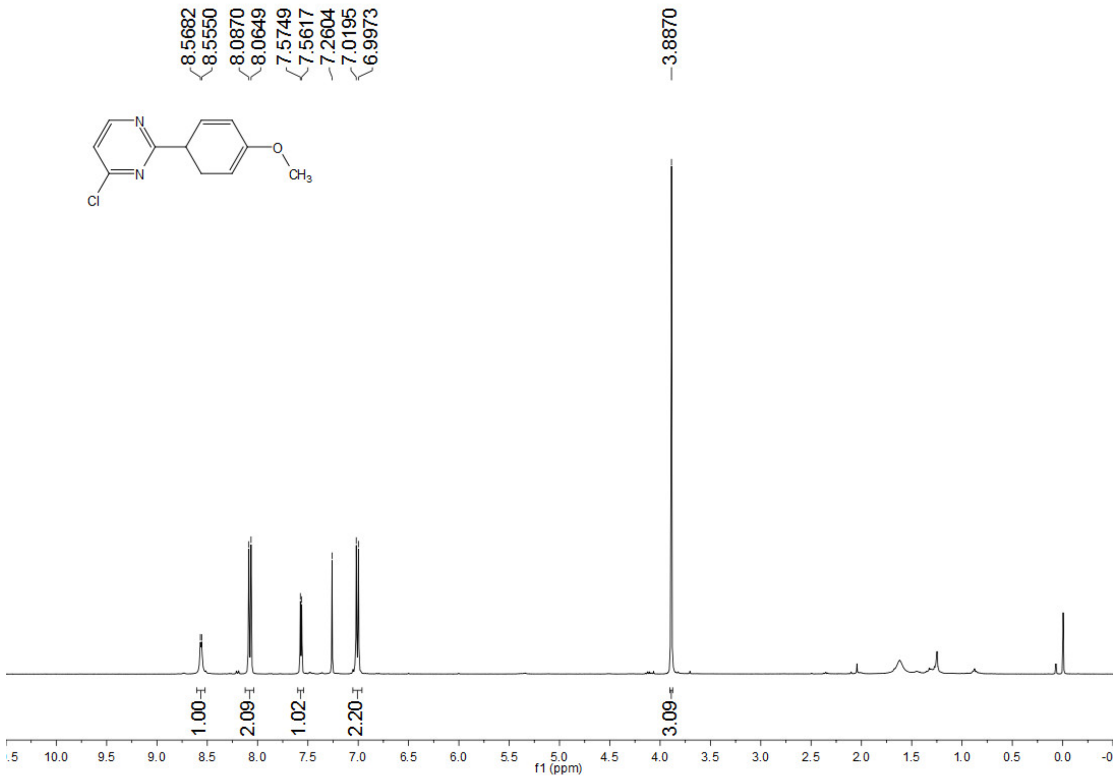

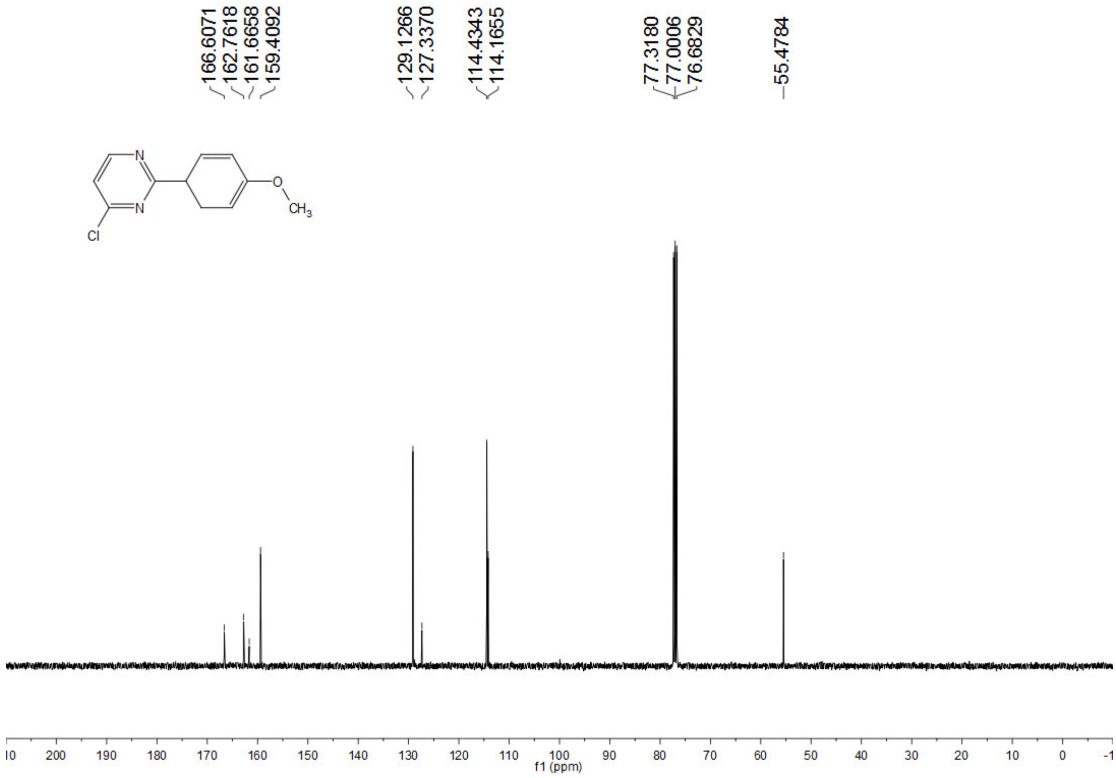

Figure S16. ^{1}H NMR and ^{13}C NMR spectra of **3l.**

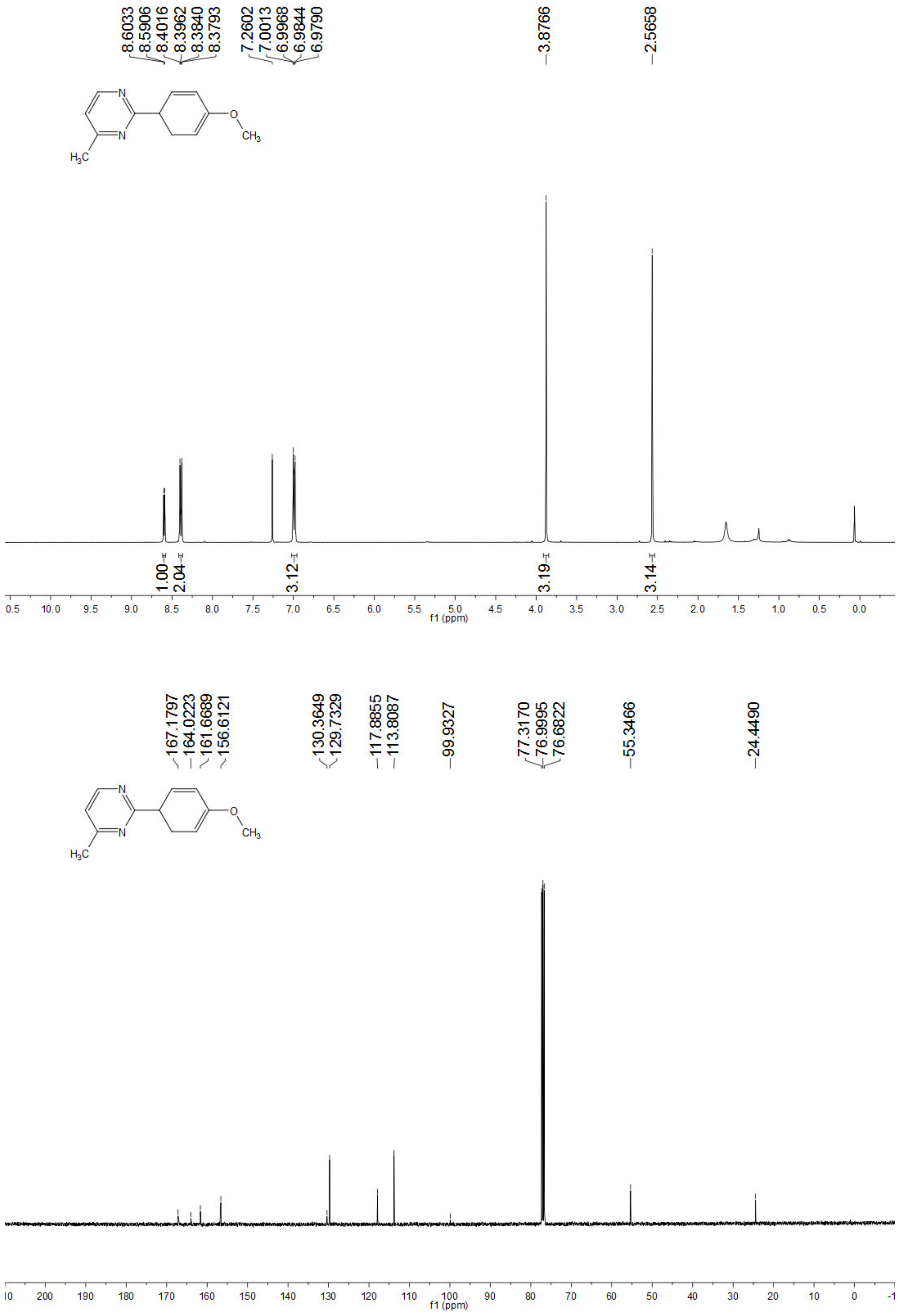

Figure S17. ^{1}H NMR and ^{13}C NMR spectra of **3m.**

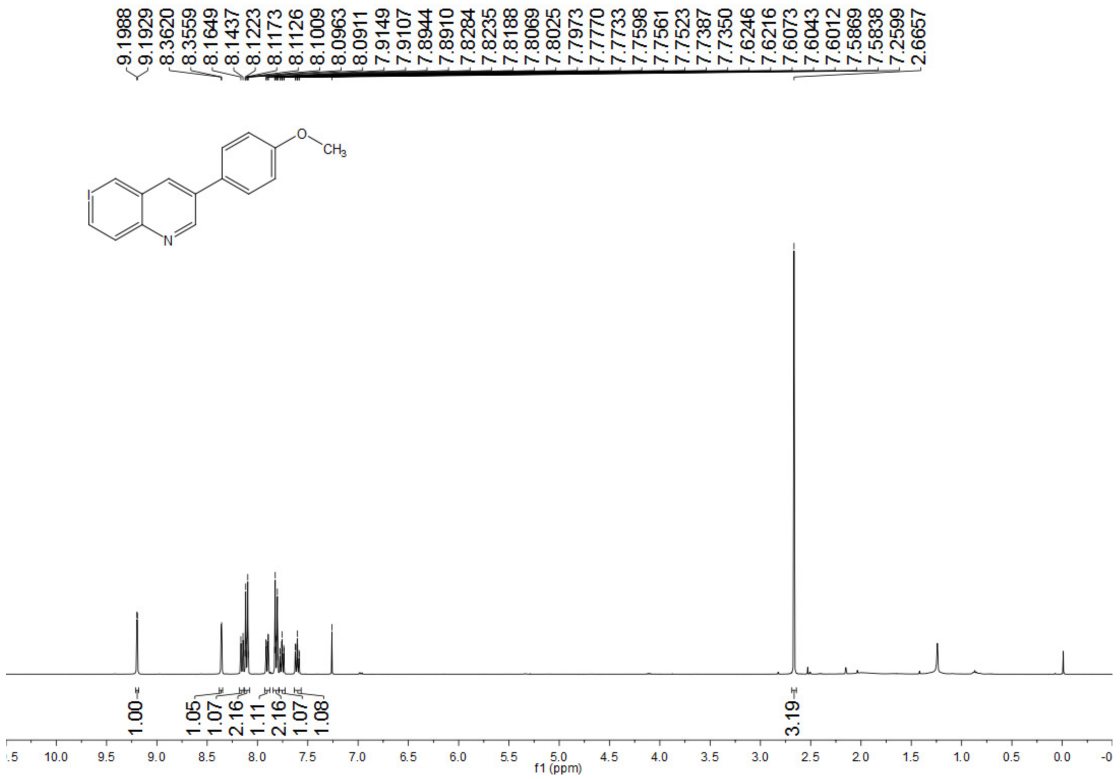

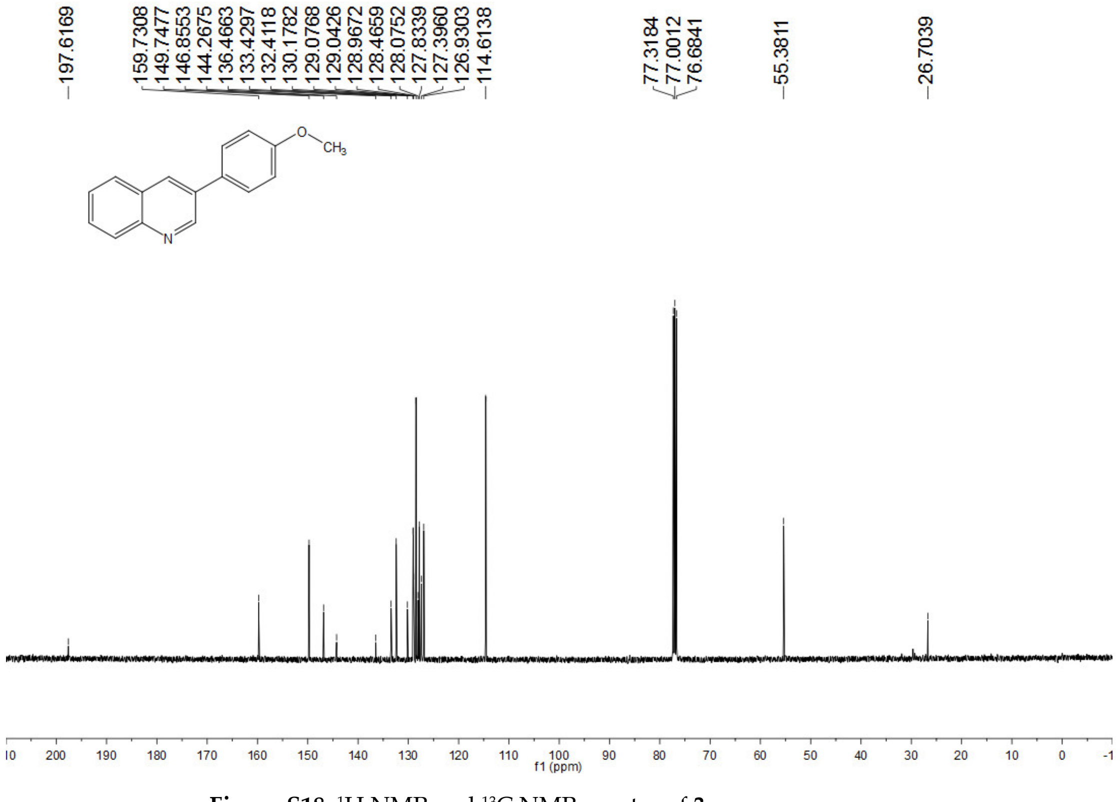

Figure S18. ^{1}H NMR and ^{13}C NMR spectra of **3n.**

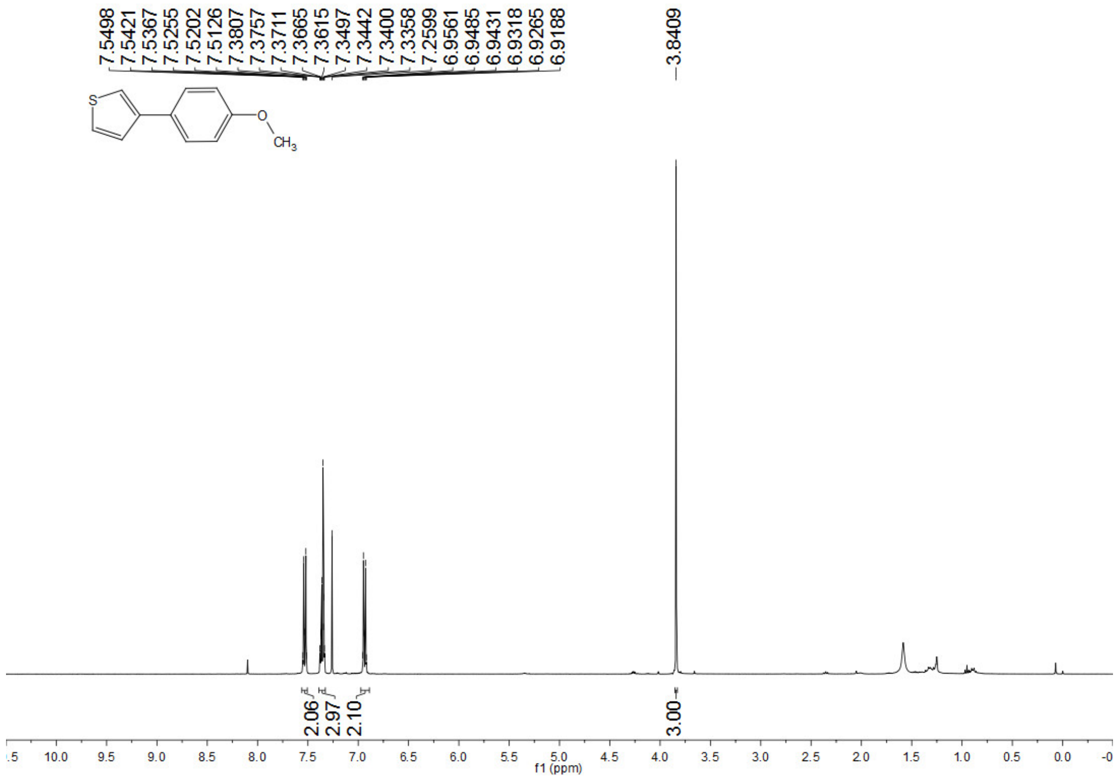

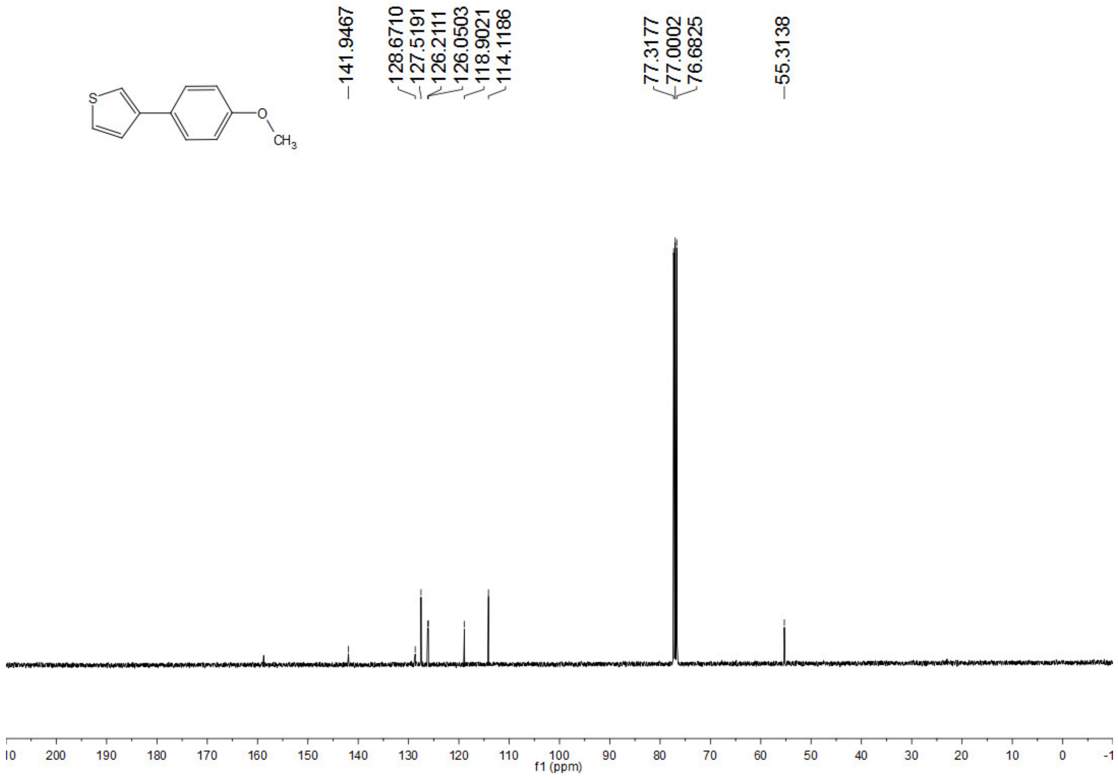

Figure S19. ^{1}H NMR and ^{13}C NMR spectra of **3o.**

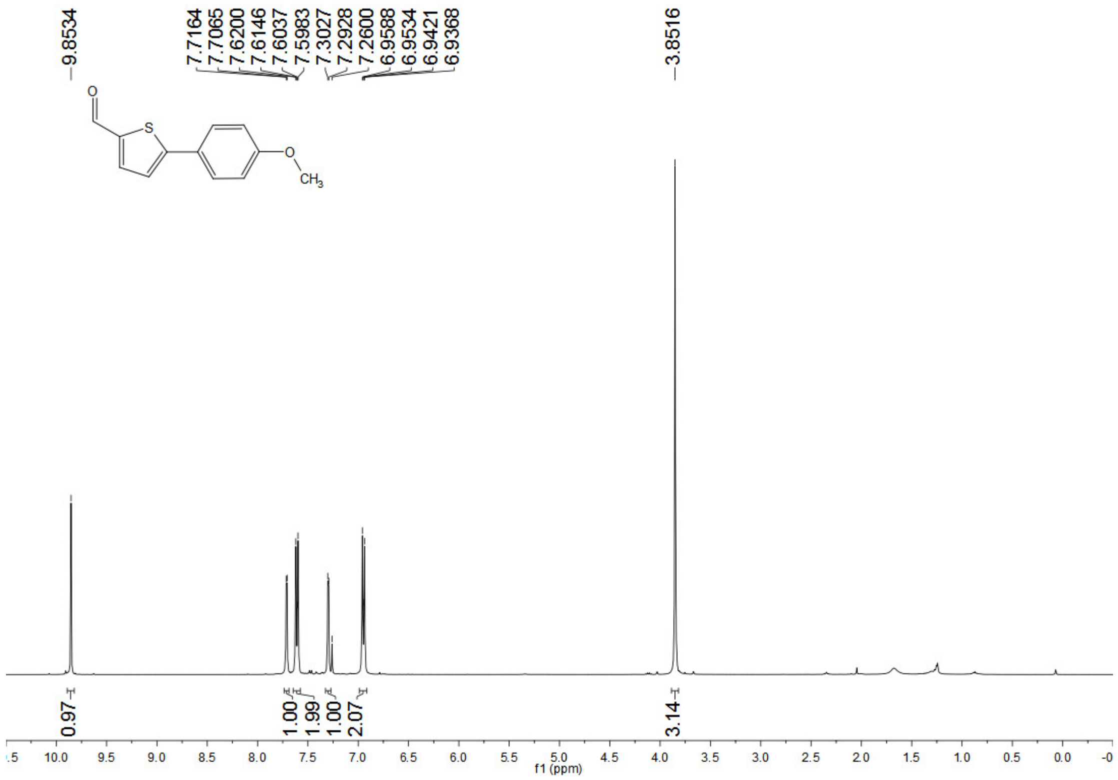

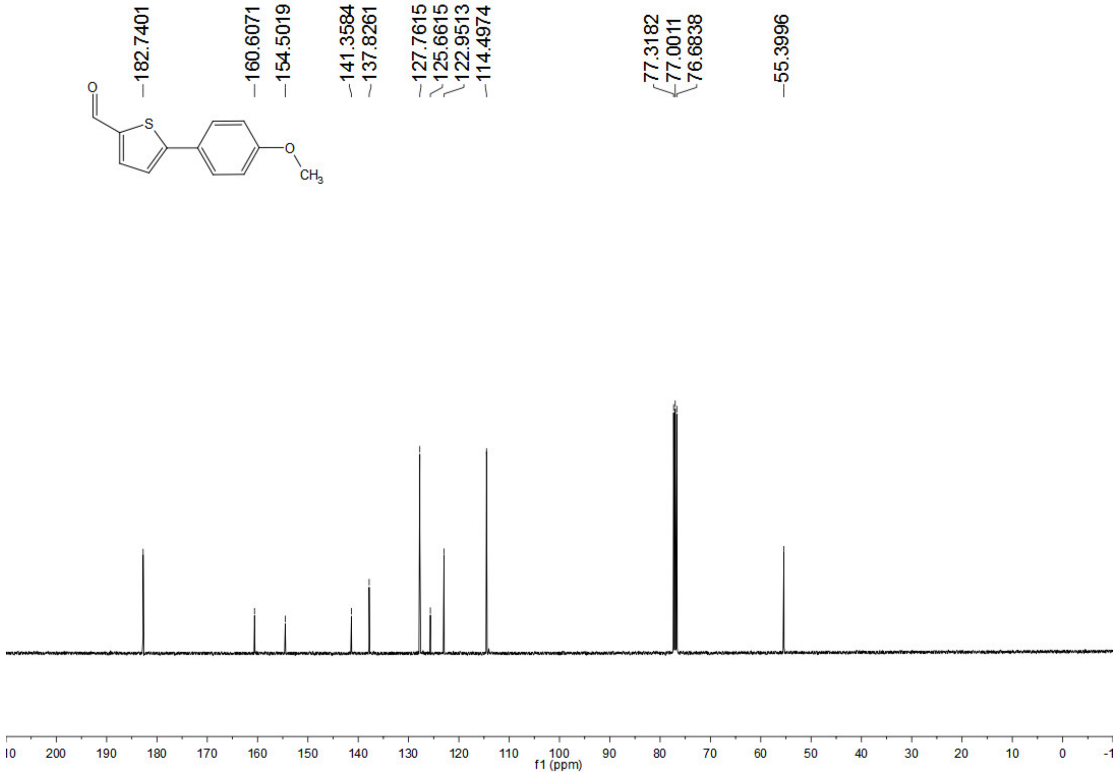

Figure S20. ^{1}H NMR and ^{13}C NMR spectra of **3p.**

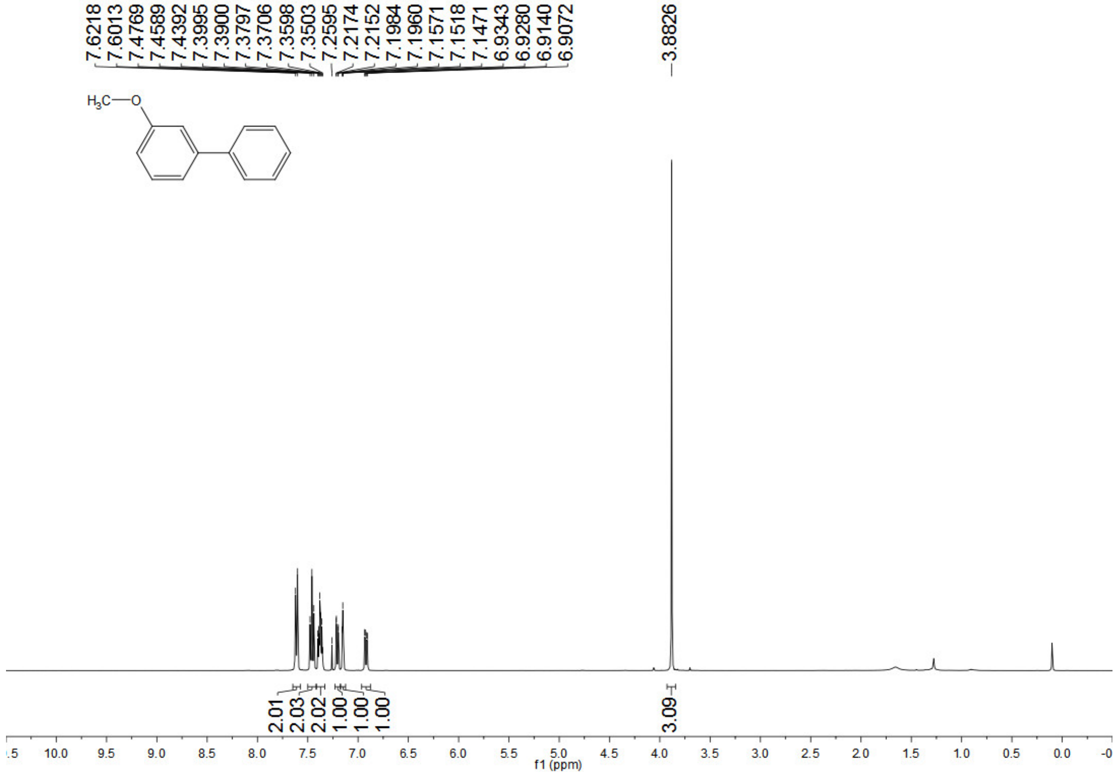

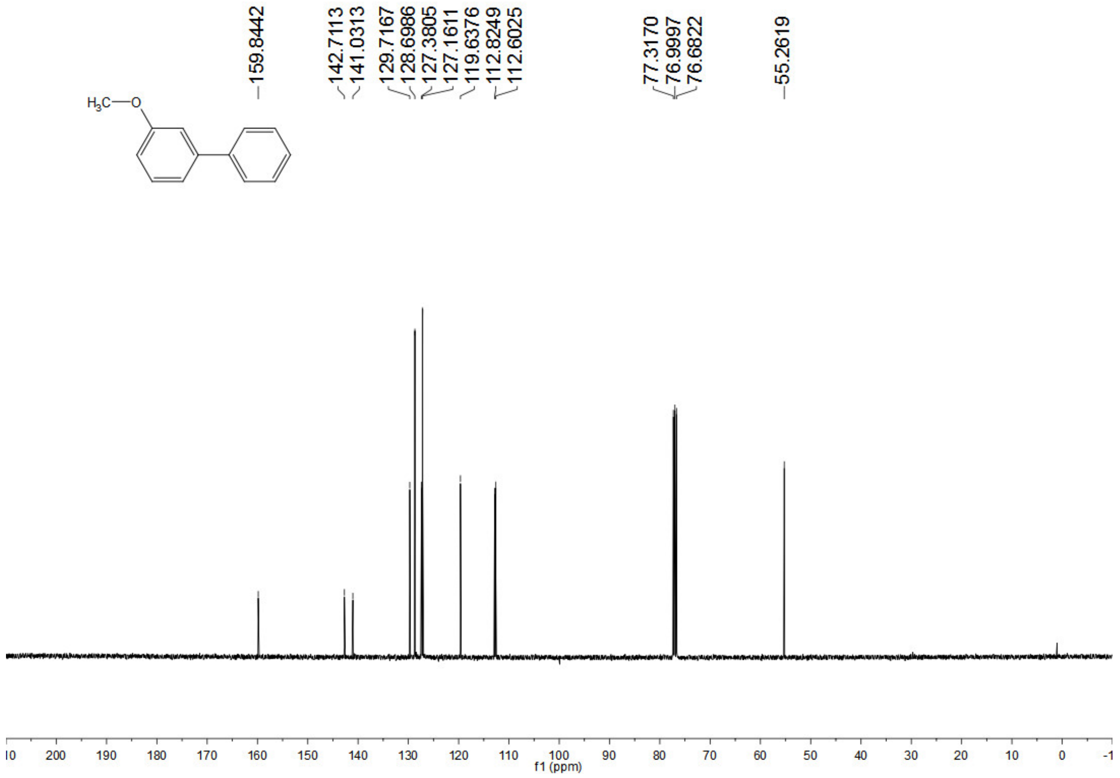

Figure S21. ^{1}H NMR and ^{13}C NMR spectra of **3q.**

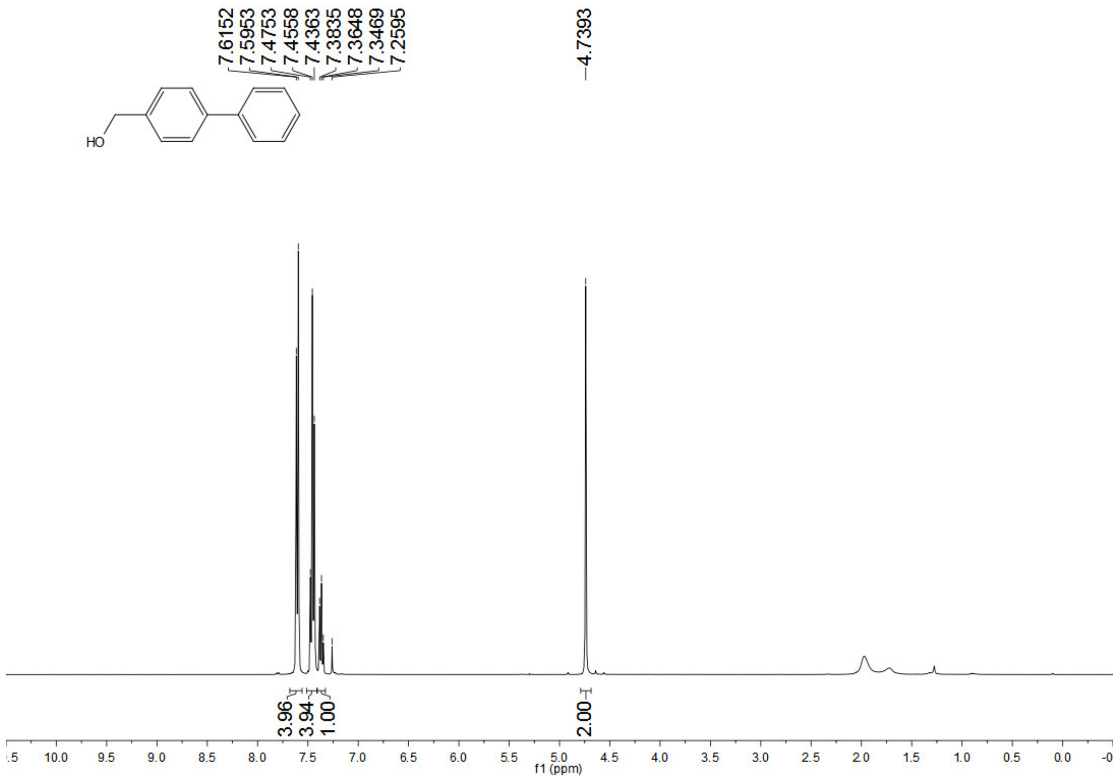

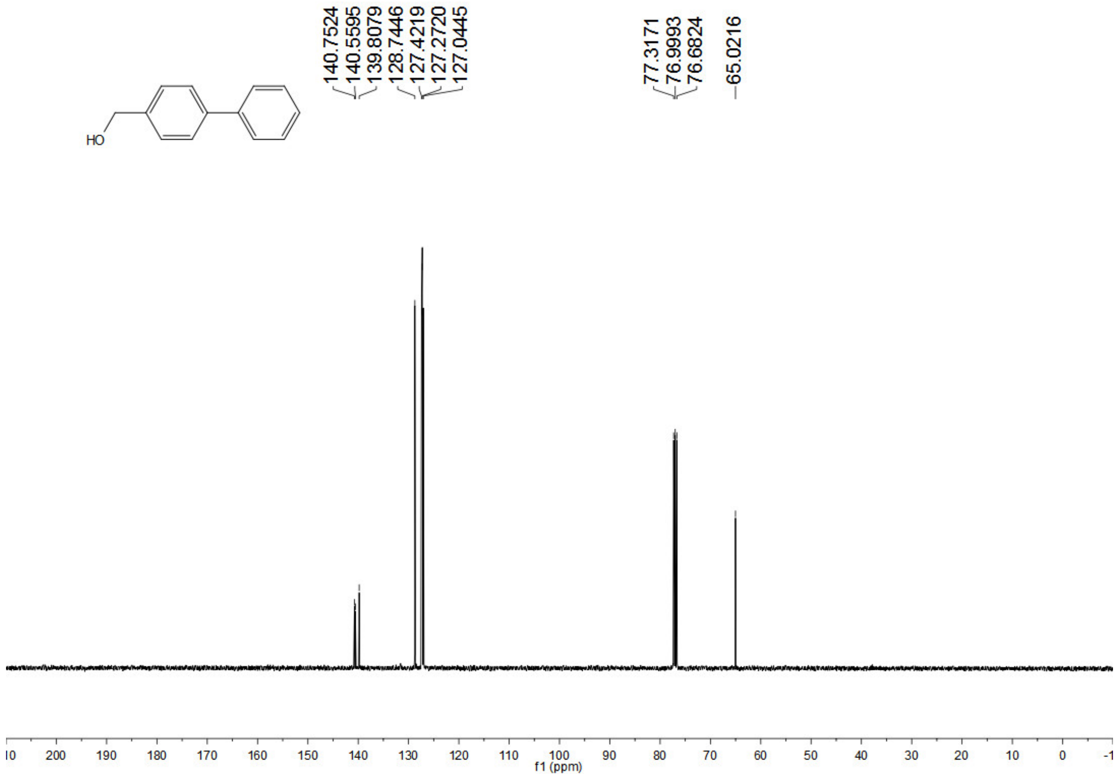

Figure S22. ^{1}H NMR and ^{13}C NMR spectra of **3r.**

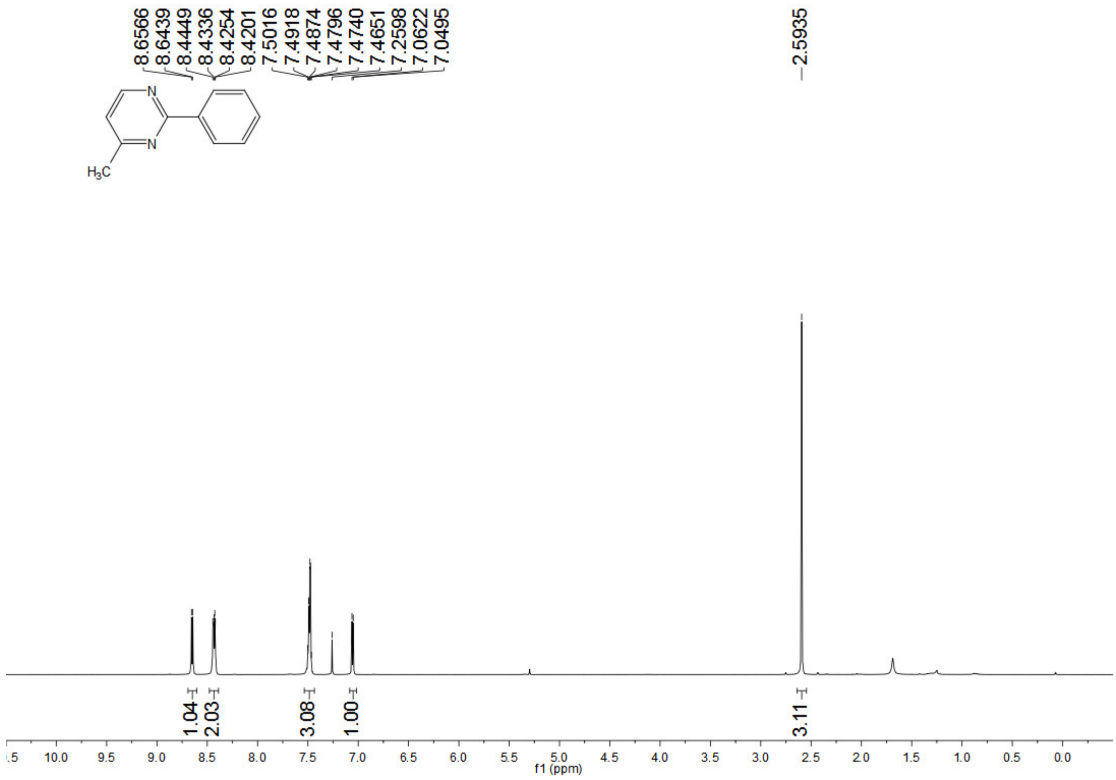

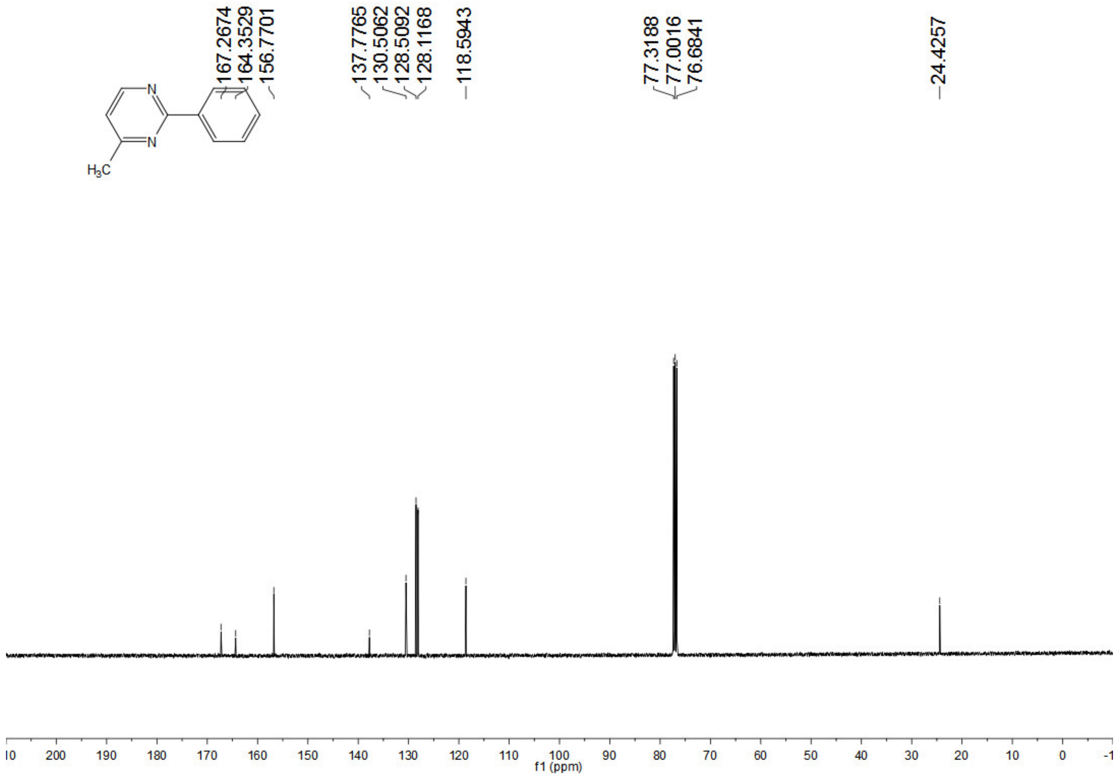

Figure S23. ^{1}H NMR and ^{13}C NMR spectra of **3s.**

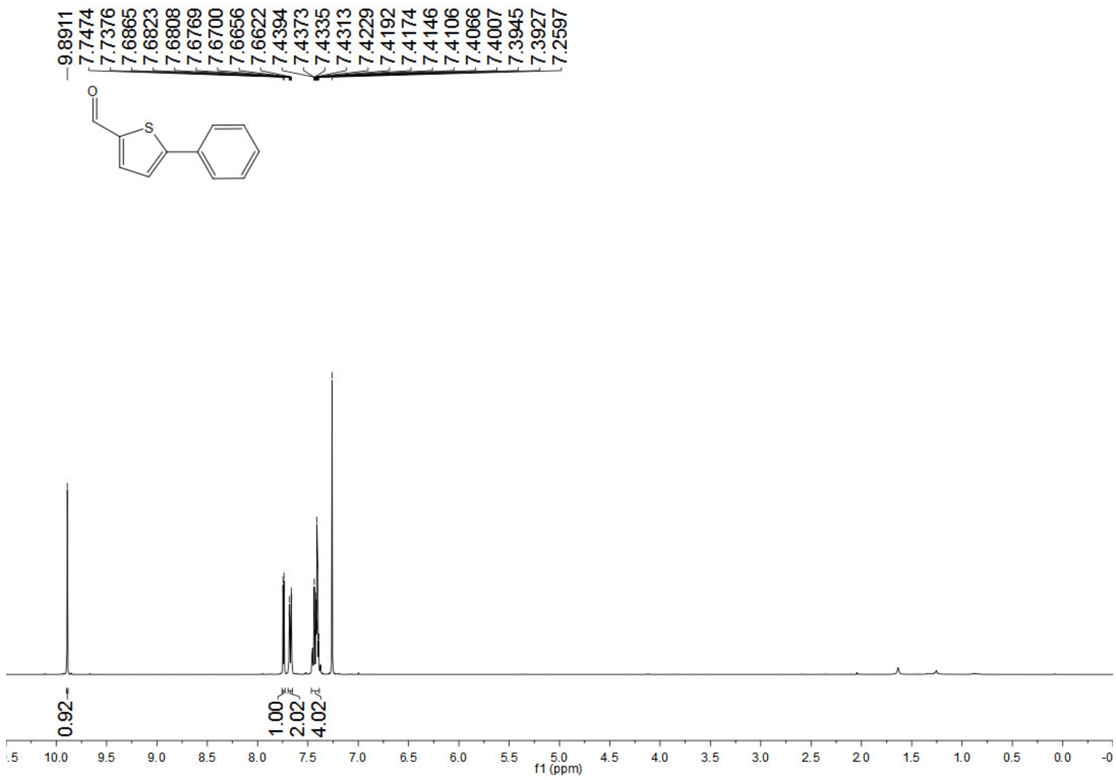

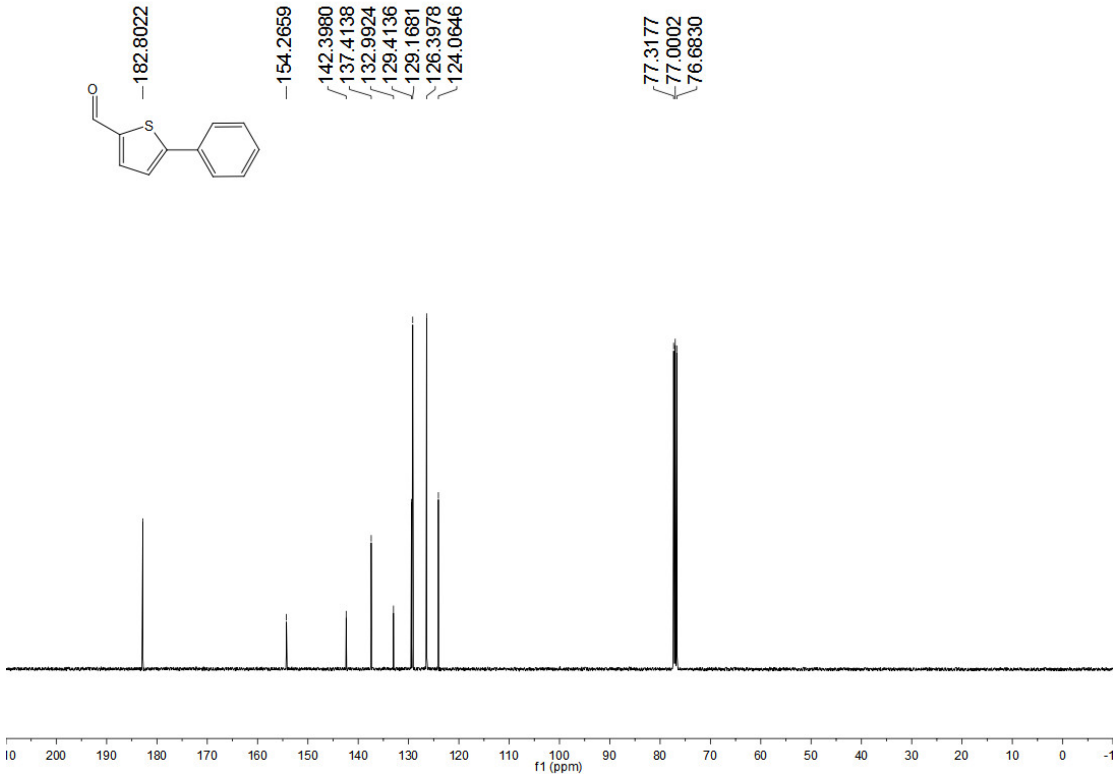

Figure S24. ^{1}H NMR and ^{13}C NMR spectra of **3t.**

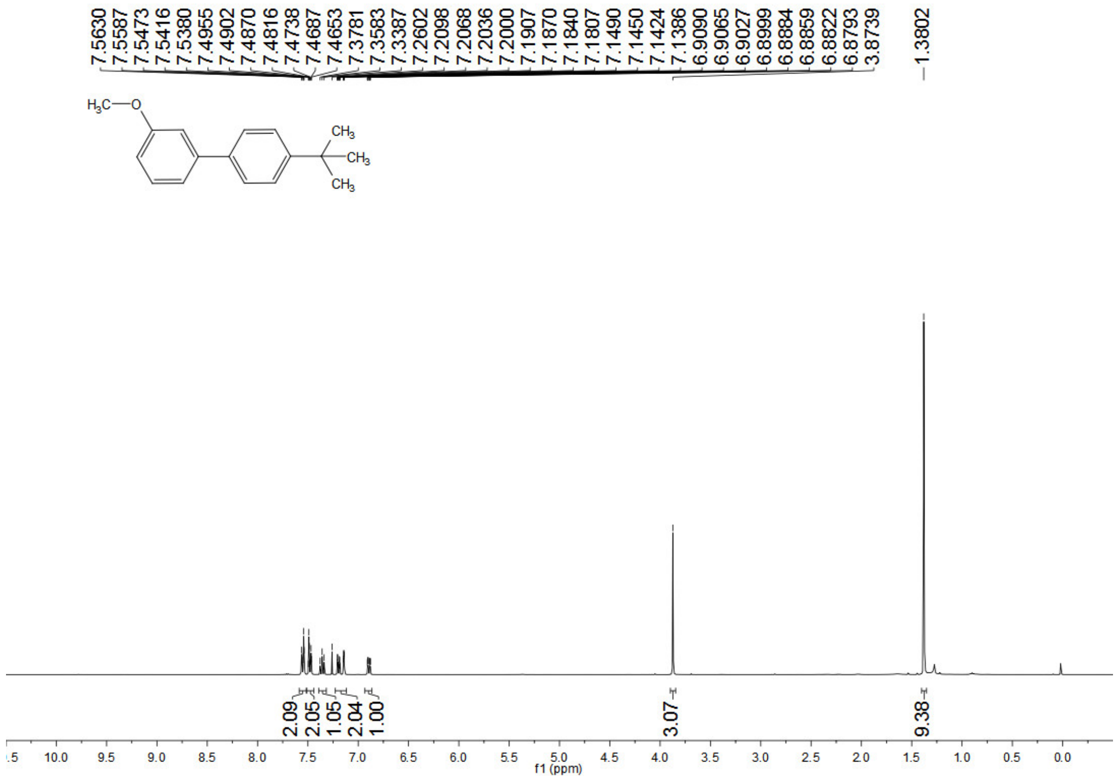

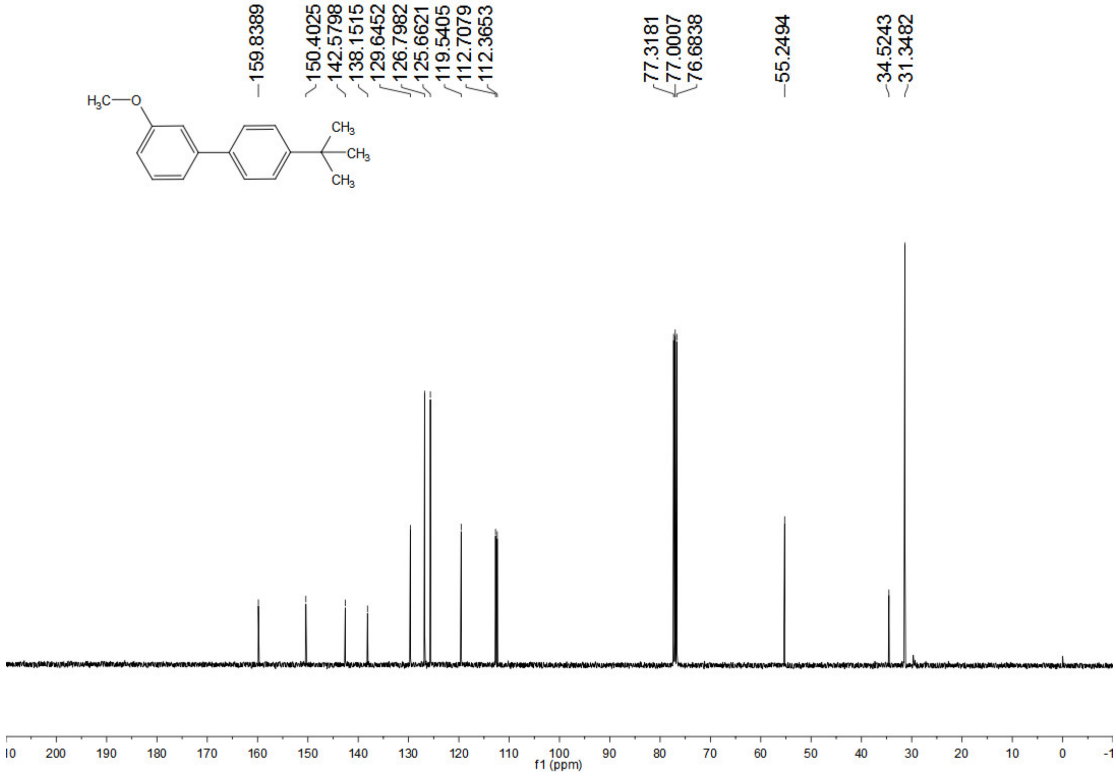

Figure S25. ^{1}H NMR and ^{13}C NMR spectra of **3u.**

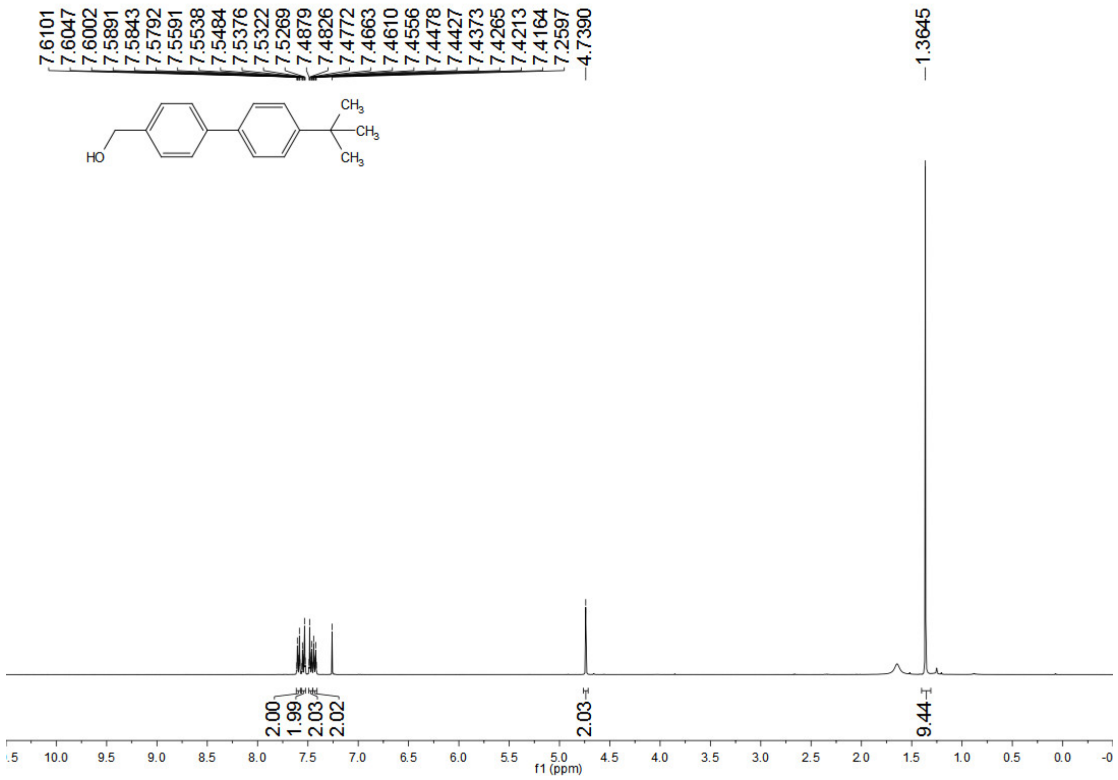

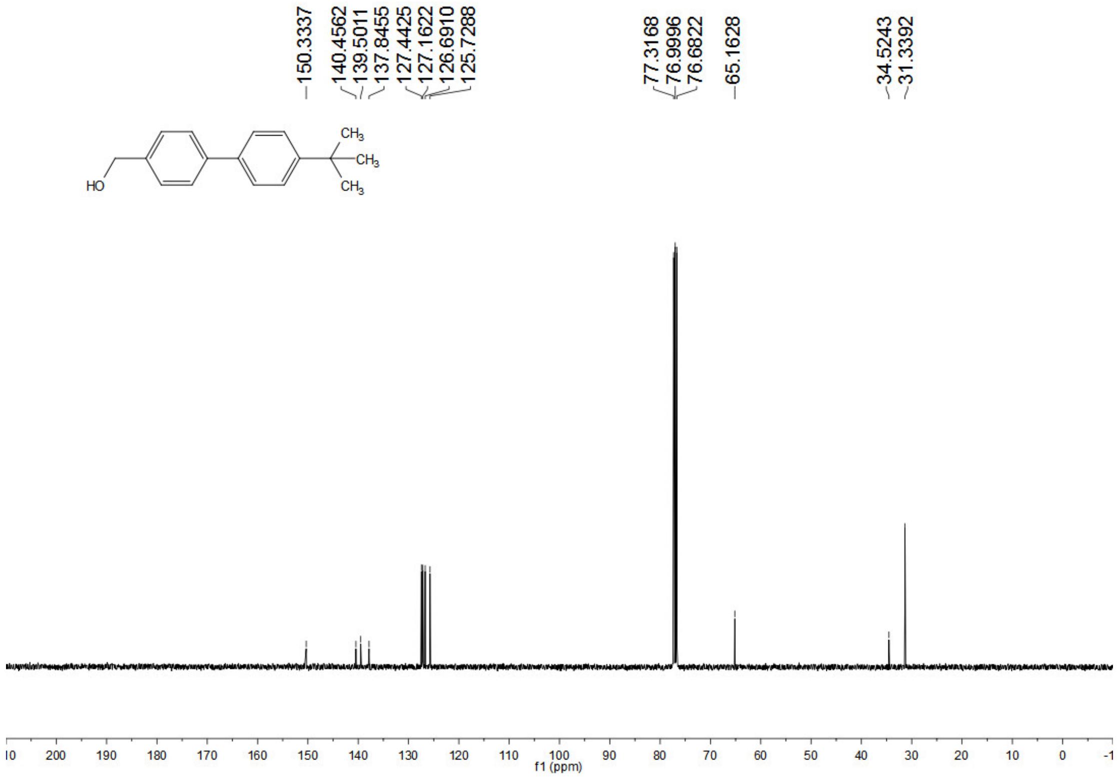

Figure S26. ^{1}H NMR and ^{13}C NMR spectra of **3v.**

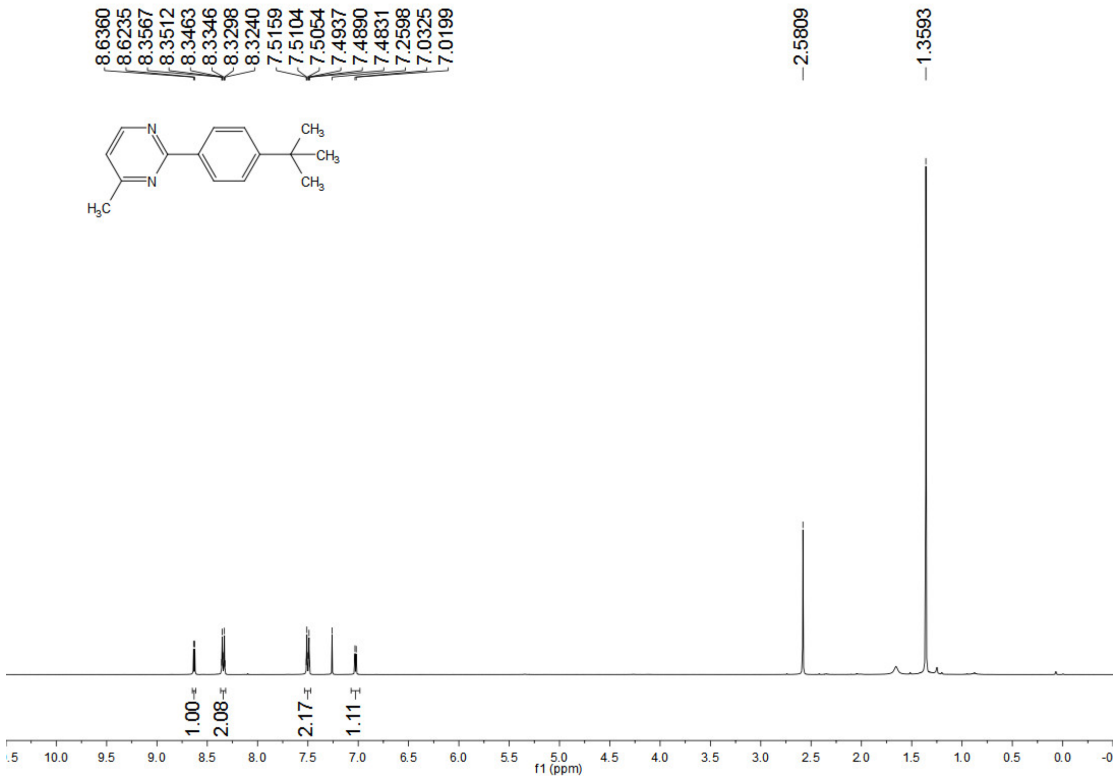

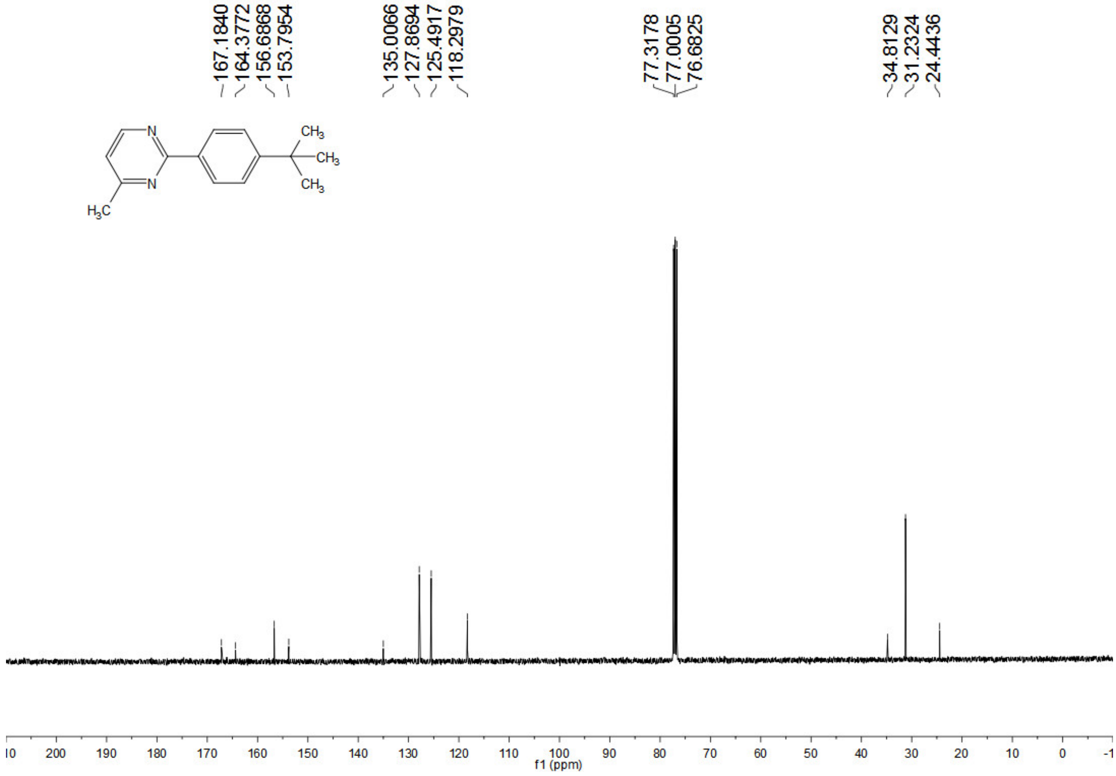

Figure S27. ^{1}H NMR and ^{13}C NMR spectra of **3w.**

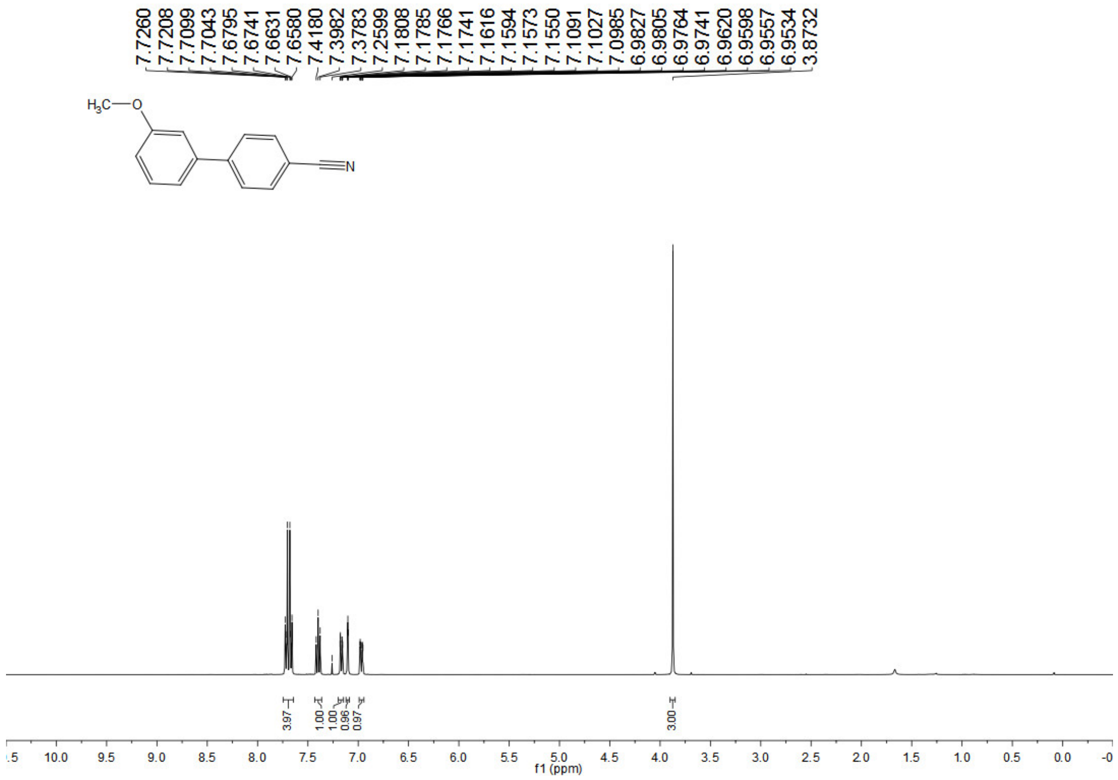

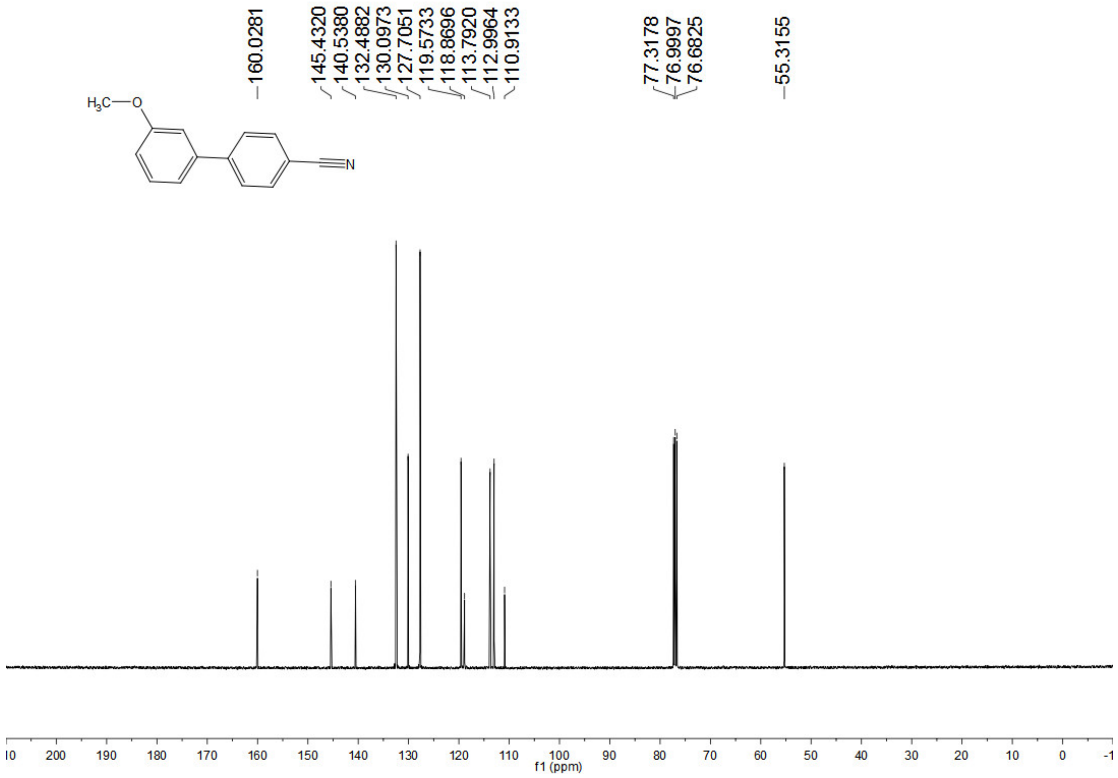

Figure S28. ^{1}H NMR and ^{13}C NMR spectra of **3x.**

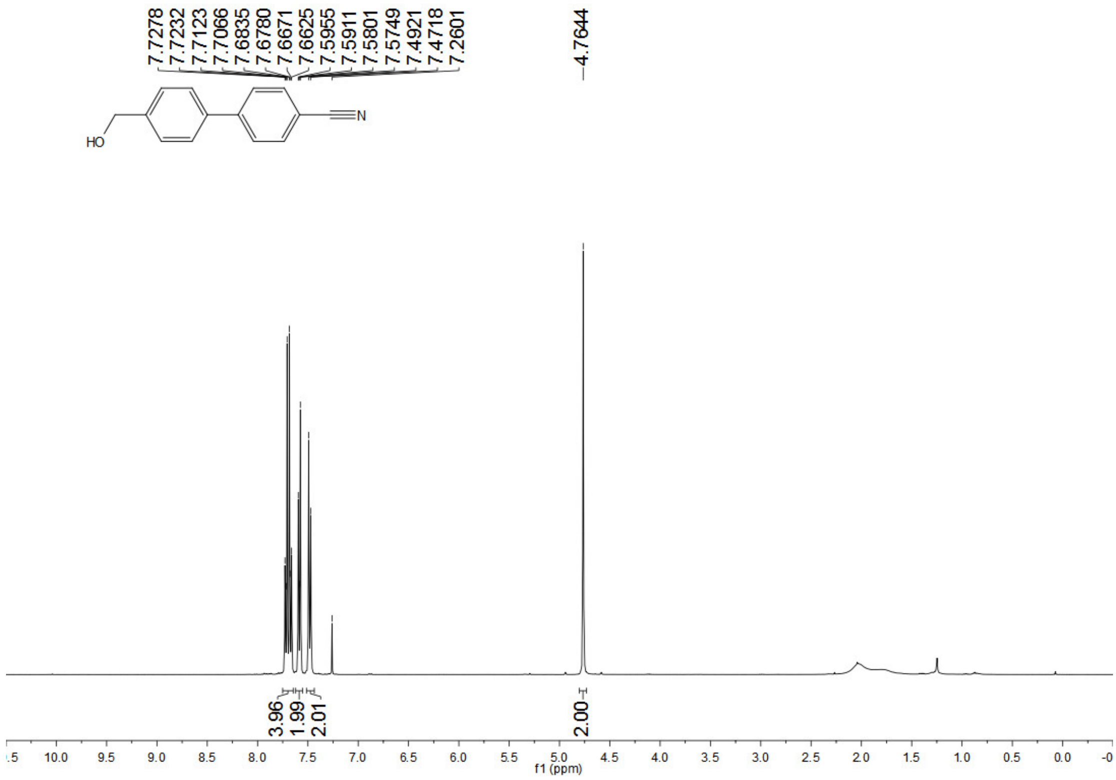

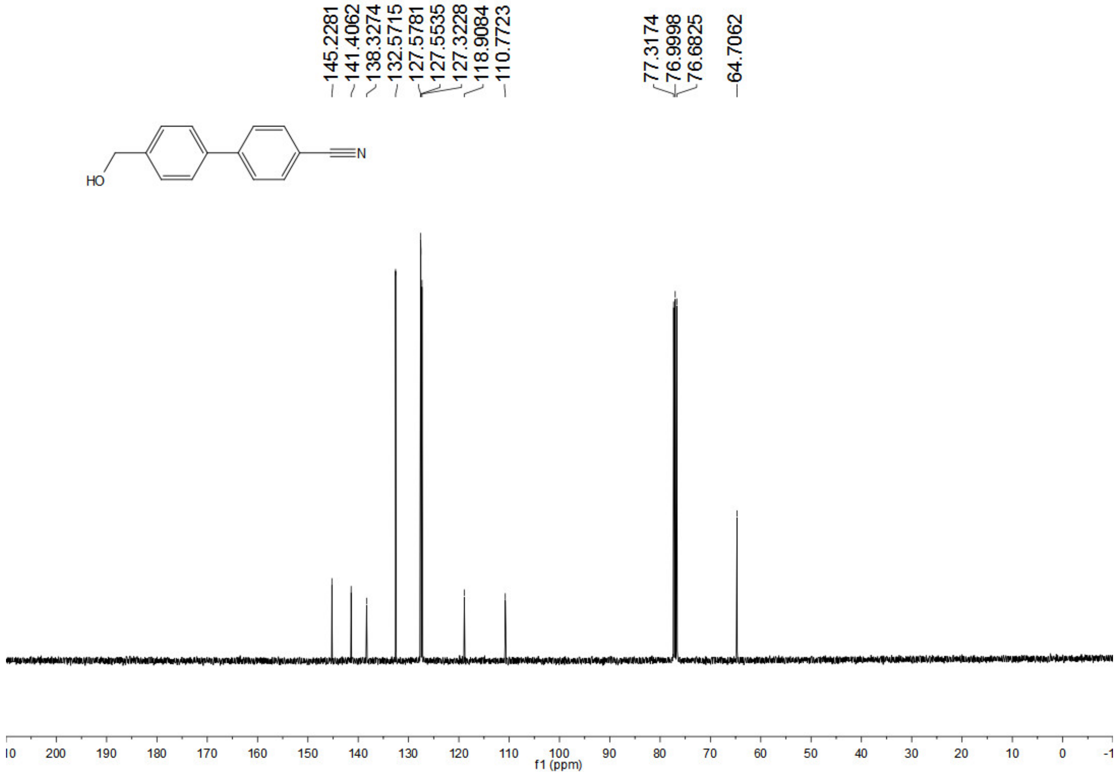

Figure S29. ^{1}H NMR and ^{13}C NMR spectra of **3y.**

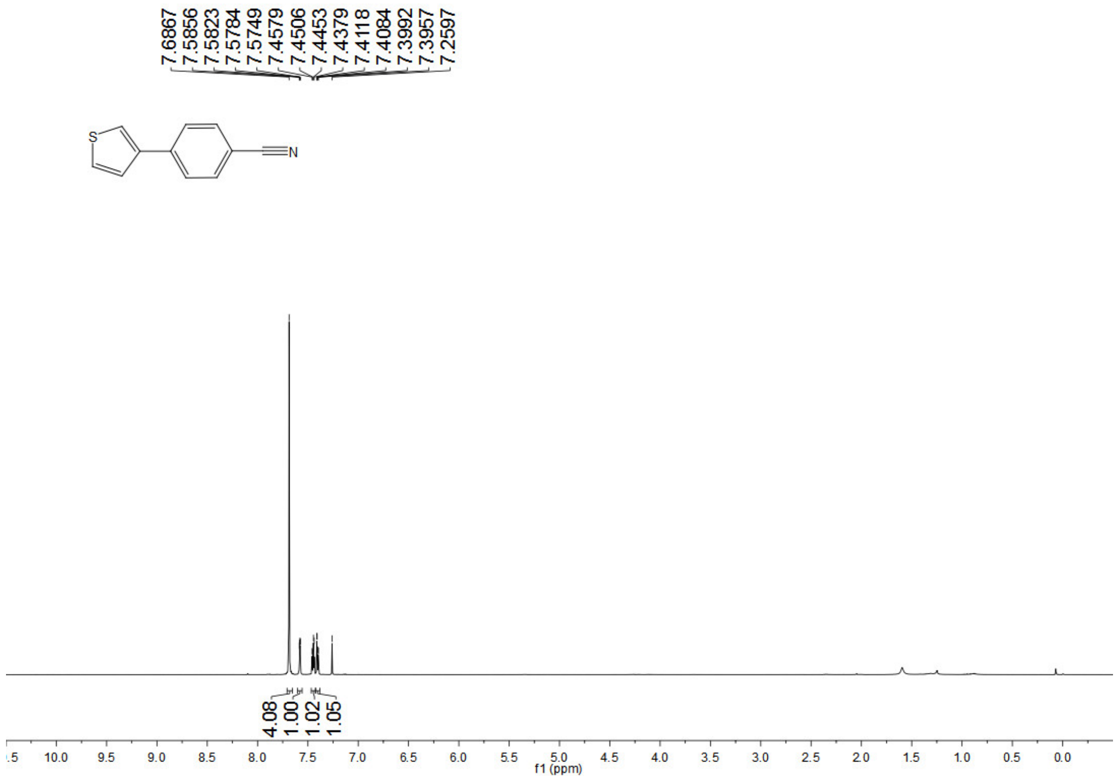

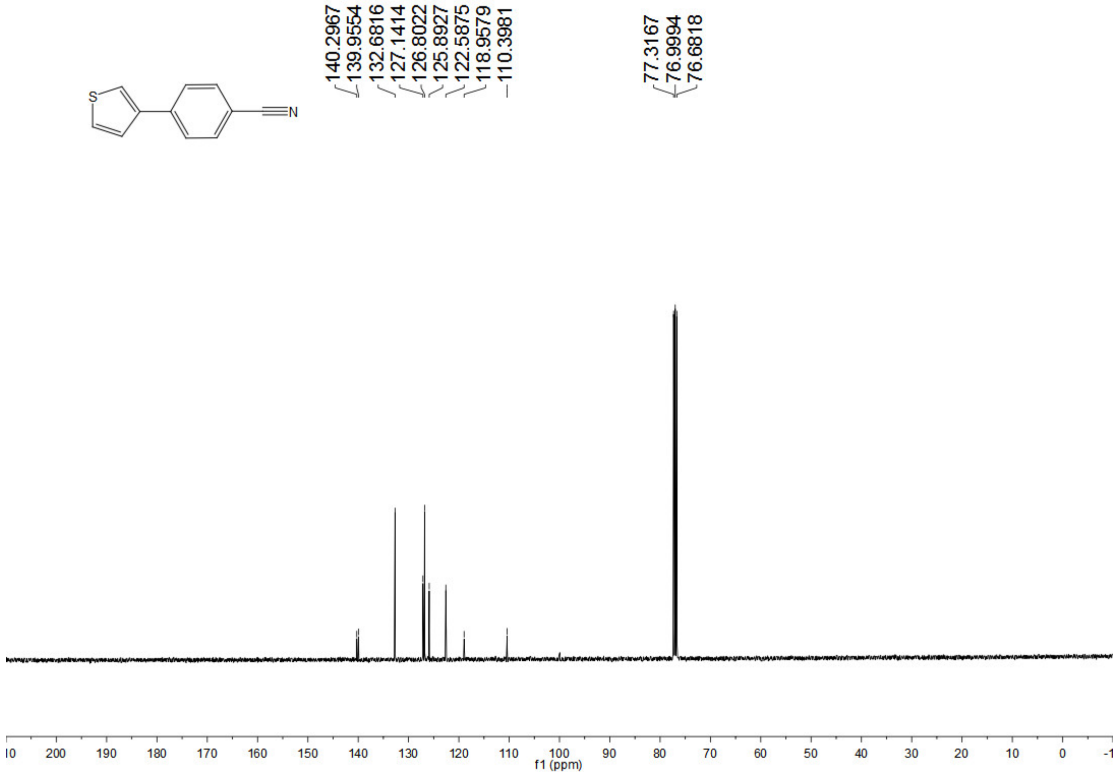

Figure S30. ^{1}H NMR and ^{13}C NMR spectra of **3z.**

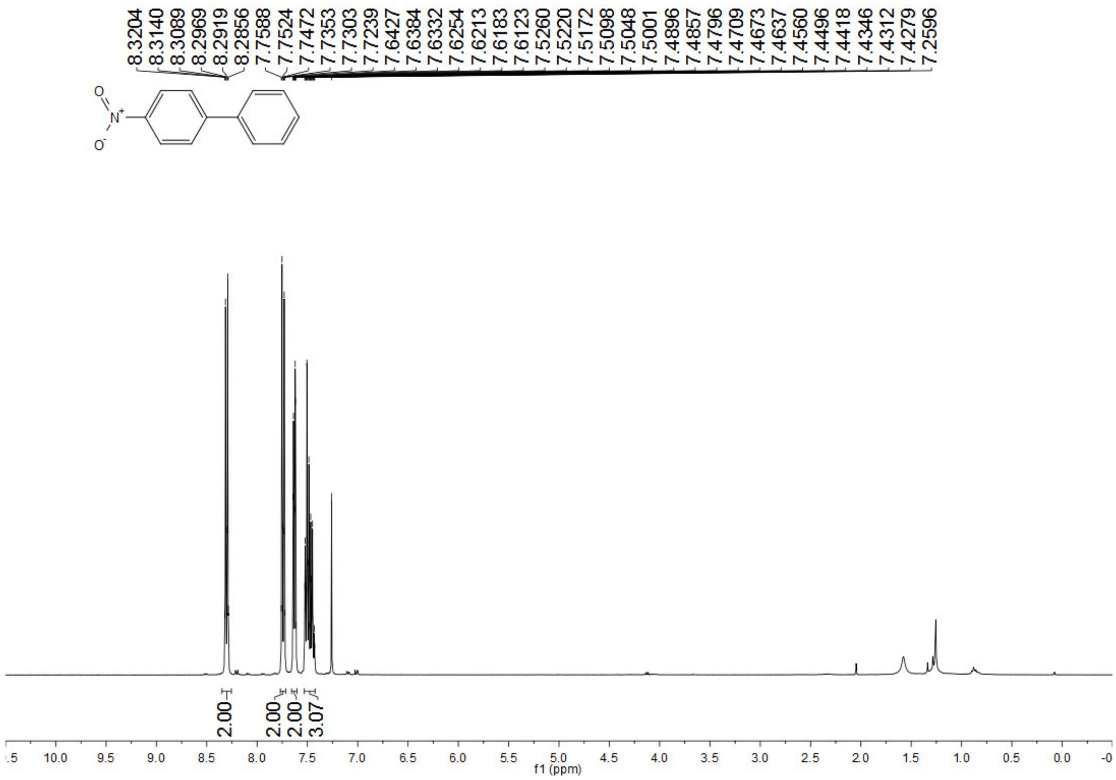

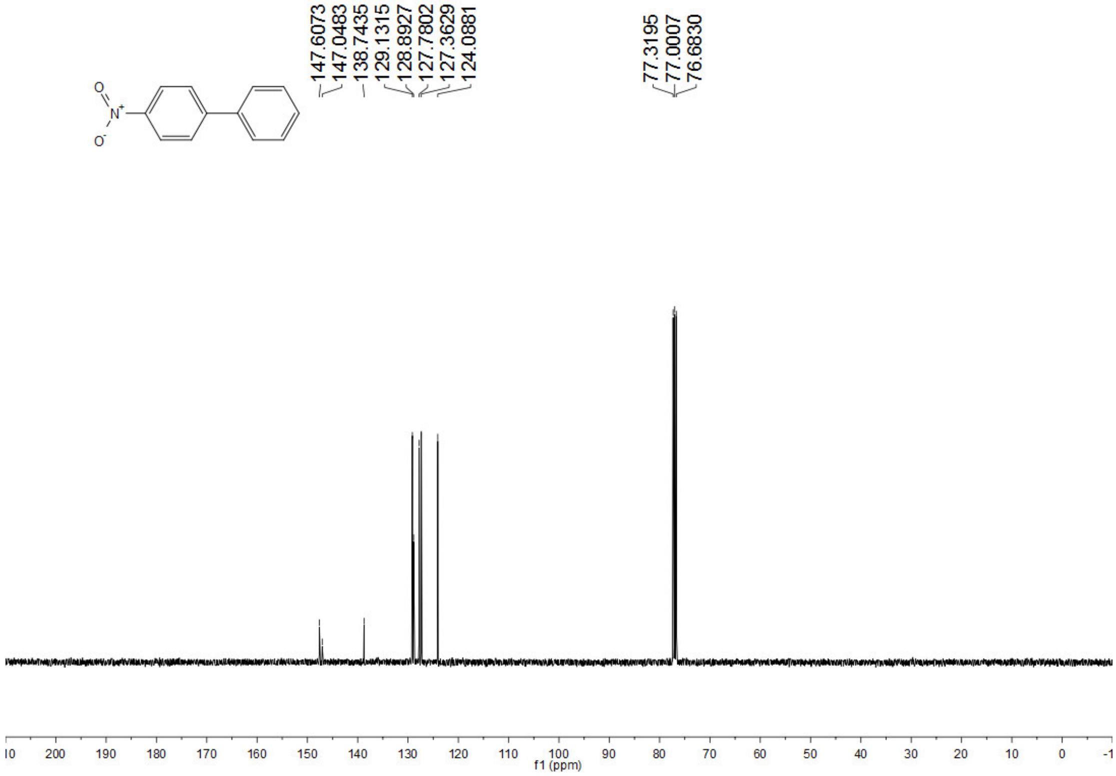

Figure S31. ^{1}H NMR and ^{13}C NMR spectra of **3aa.**

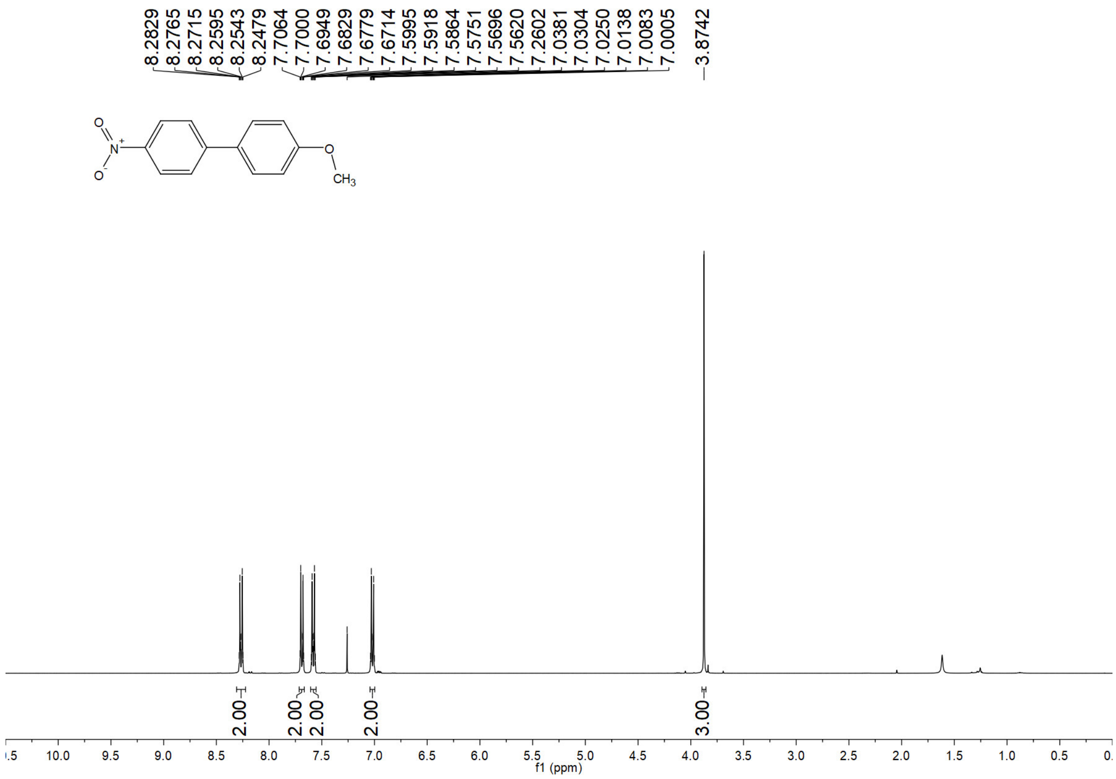

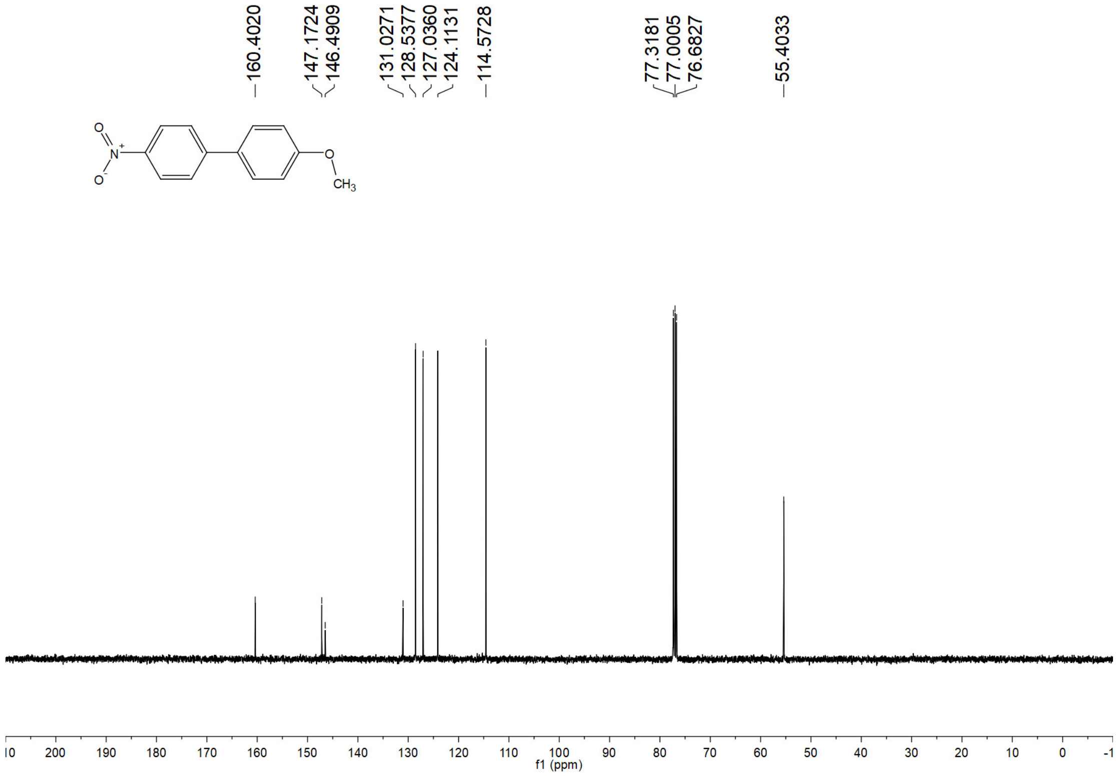

Figure S32. ^{1}H NMR and ^{13}C NMR spectra of **3ab.**
